# Integrated Activity and Genetic Profiling of Secreted Peptidases in *Cryptococcus neoformans* Reveals an Aspartyl Peptidase Required for Low pH Survival and Virulence

**DOI:** 10.1371/journal.ppat.1006051

**Published:** 2016-12-15

**Authors:** Starlynn C. Clarke, Phillip A. Dumesic, Christina M. Homer, Anthony J. O’Donoghue, Florencia La Greca, Lenka Pallova, Pavel Majer, Hiten D. Madhani, Charles S. Craik

**Affiliations:** 1 Department of Pharmaceutical Chemistry, University of California, San Francisco, San Francisco, California, United States of America; 2 Department of Biochemistry and Biophysics, University of California, San Francisco, San Francisco, California, United States of America; 3 Institute of Organic Chemistry and Biochemistry, Academy of Sciences of the Czech Republic, Prague, Czech Republic; University of Birmingham, UNITED KINGDOM

## Abstract

The opportunistic fungal pathogen *Cryptococcus neoformans* is a major cause of mortality in immunocompromised individuals, resulting in more than 600,000 deaths per year. Many human fungal pathogens secrete peptidases that influence virulence, but in most cases the substrate specificity and regulation of these enzymes remains poorly understood. The paucity of such information is a roadblock to our understanding of the biological functions of peptidases and whether or not these enzymes are viable therapeutic targets. We report here an unbiased analysis of secreted peptidase activity and specificity in *C*. *neoformans* using a mass spectrometry-based substrate profiling strategy and subsequent functional investigations. Our initial studies revealed that global peptidase activity and specificity are dramatically altered by environmental conditions. To uncover the substrate preferences of individual enzymes and interrogate their biological functions, we constructed and profiled a ten-member gene deletion collection of candidate secreted peptidases. Through this deletion approach, we characterized the substrate specificity of three peptidases within the context of the *C*. *neoformans* secretome, including an enzyme known to be important for fungal entry into the brain. We selected a previously uncharacterized peptidase, which we term **M**ajor **a**spart**y**l peptidase 1 (May1), for detailed study due to its substantial contribution to extracellular proteolytic activity. Based on the preference of May1 for proteolysis between hydrophobic amino acids, we screened a focused library of aspartyl peptidase inhibitors and identified four high-affinity antagonists. Finally, we tested *may1Δ* strains in a mouse model of *C*. *neoformans* infection and found that strains lacking this enzyme are significantly attenuated for virulence. Our study reveals the secreted peptidase activity and specificity of an important human fungal pathogen, identifies responsible enzymes through genetic tests of their function, and demonstrates how this information can guide the development of high affinity small molecule inhibitors.

## Author Summary

Many pathogenic organisms secrete peptidases. The activity of these enzymes often contributes to virulence, making their study crucial for understanding host-pathogen biology and developing therapeutics. In this report, we employed an unbiased, activity-based profiling assay to examine the secreted peptidases of a fungal pathogen, *Cryptococcus neoformans*, which is responsible for 40% of AIDS-related deaths. We discovered which peptidases are secreted, identified their substrate specificity, and interrogated their biological functions. Through this analysis, we identified a principal enzyme responsible for the extracellular peptidase activity of *C*. *neoformans*, May1, and demonstrated its importance for growth in acidic environments. Characterization of its substrate preferences allowed us to identify compounds that are potent substrate-based inhibitors of May1 activity. Finally, we found that the presence of this enzyme promotes virulence in a mouse model of infection. Our comprehensive study reveals the expression, regulation and function of *C*. *neoformans* secreted peptidases, including evidence for the role of a novel aspartyl peptidase in virulence.

## Introduction

*Cryptococcus neoformans* is an opportunistic fungal pathogen responsible for 40% of all AIDS-related deaths [1,2]. Of the one million new infections occurring worldwide annually, greater than 60% result in death due to the limited efficacy and availability of therapeutics [3]. Only three classes of drugs are currently approved for treatment of fungal infections, thus there is a significant need for development of new antifungal compounds [3–5].

Peptidases are secreted by many types of pathogens including bacteria, fungi and parasites and often serve critical roles related to survival and virulence [6–11]. Direct targeting of peptidases expressed by pathogenic organisms has proven to be a successful therapeutic strategy, notably in the development of Hepatitis C Virus (HCV) and Human Immunodeficiency Virus (HIV) protease inhibitors [12,13]. Additionally, the identification and characterization of peptidases secreted by pathogens have contributed to the formulation of new diagnostic approaches based on detection of these proteolytic activities [14–16].

Pathogenic fungi express extracellular peptidases for wide-ranging functions including host tissue invasion, nutrient acquisition and regulation of mating [17–19]. A single organism may simultaneously secrete multiple peptidases with divergent substrate specificities and requirements for activity that are tailored to their biological functions. In addition, peptidase secretion and activation are often stimulated by extracellular conditions, as distinct proteolytic functions can be important for different environments. *Candida albicans* and *Aspergillus fumigatus*, two prominent fungal pathogens, each secrete several peptidases with defined roles in virulence, while dermatophytes and the causative agent of white-nose syndrome *Pseudogymnoascus destructans* use extracellular peptidases to degrade host tissues [20–26]. Multiple peptidases have been identified in the secreted proteome of *C*. *neoformans*, including a metallopeptidase that is required for dissemination to the central nervous system (CNS) in a mouse infection model [27–34]. Interestingly, the level of peptidase secretion has been shown to vary between isolates in *Cryptococcus* species and in many cases higher secretion has been correlated with increased virulence [35–38]. Although these findings suggest that extracellular peptidases are involved in *C*. *neoformans* pathogenicity, the delineation of their functions and their validation as therapeutic targets is limited by poor understanding of their activity, specificity and regulation.

In this work, we used a comprehensive activity-based approach to characterize secreted peptidases in *C*. *neoformans* culture supernatants. This strategy, termed Multiplex Substrate Profiling by Mass Spectrometry (MSP-MS), relies on mass spectrometry to identify cleavage events within a defined 228-member library comprising physiochemically diverse tetradecapeptides [39]. The scope and design of the library allows detection of cleavage events from multiple peptidases simultaneously, and the resulting data are informative for understanding activity on both a global and individual enzyme level. Activity-based profiling stands in contrast to traditional proteomics methods that catalog which peptidases are present but do not provide information on how each enzyme contributes to the overall proteolytic activity [11,27]. Likewise, candidate-based approaches focusing on single proteolytic activities isolated from cultures may not accurately represent how these enzymes function within the secreted peptidase milieu [31,32].

To investigate the secreted peptidases of *C*. *neoformans* and test the influence of environment on global proteolytic activity, we cultured fungal cells under two different conditions and then isolated the cell-free supernatants for substrate specificity profiling. These experiments revealed that overall peptidase specificity differs greatly in response to extracellular conditions. To uncover the contribution of individual enzymes to the total proteolytic activity, ten candidate peptidases were individually deleted and conditioned media generated from each mutant strain was compared to the parental strain. Through this approach, we identified and defined the putative substrate preferences of three peptidases, including a previously uncharacterized secreted aspartyl peptidase. We found that this enzyme is the dominant contributor to extracellular endopeptidase activity at acidic pH and determined that this activity is required for tolerance to low pH environments. Analysis of its substrate specificity enabled us to screen an appropriately focused library of aspartyl peptidase inhibitors, which led to the identification of potent *in vitro* antagonists. Finally, we found that deletion strains of this enzyme are attenuated for virulence in a mouse inhalation model of *C*. *neoformans* infection.

Our in-depth characterization of extracellular peptidases in *C*. *neoformans* establishes a framework for uncovering the biological functions of these enzymes. As demonstrated by our identification of a peptidase required for virulence, examining the roles of these enzymes is critical to understanding the pathogenicity of *C*. *neoformans*. Furthermore, the methods described here are applicable to the discovery and characterization of secreted peptidases from other pathogenic organisms.

## Results

### Global secreted peptidase profiling in *C*. *neoformans* reveals abundant activity and environment-dependent specificity

**Fig 1:**
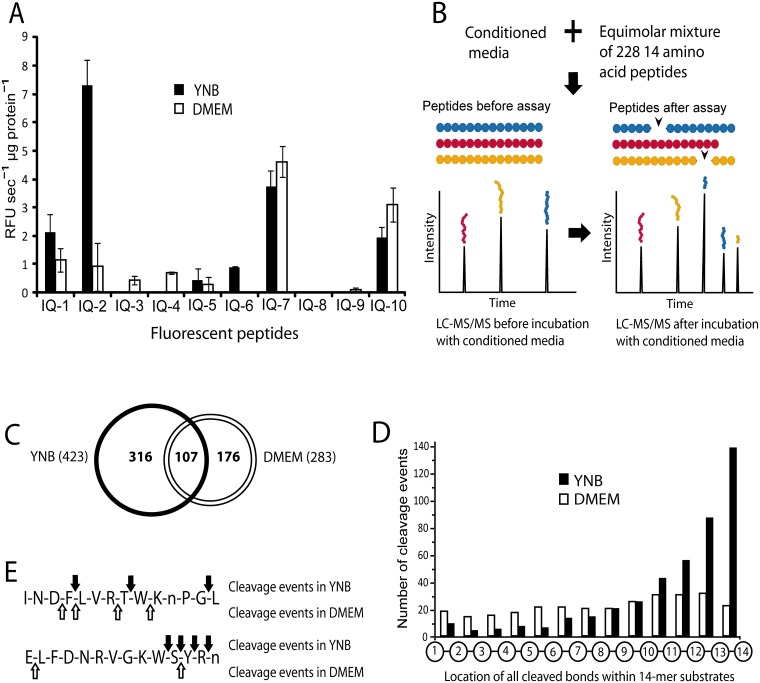
Profiling of *C*. *neoformans* conditioned media reveals abundant secreted peptidase activity with environment-specific regulation. (A) Profiling of secreted peptidase activity present in YNB or DMEM media conditioned by wild-type cells using a panel of internally quenched (IQ) fluorescent peptides. Columns represent mean ± S.D. (B) Schematic of Multiplex Substrate Profiling by Mass Spectrometry (MSP-MS). Conditioned media is combined with a 228-member peptide library and mass spectrometry analysis is run before and after incubation to identify cleavage events. Norleucine replaces methionine in the MSP-MS library and is indicated by an “n” in representations of the peptides. (C) Several hundred cleavage events were detected in both YNB and DMEM conditioned media profiled by MSP-MS. (D) Positional profiling of all cleaved bonds detected by MSP-MS in either media type. (E) Two representative examples of peptides cleaved in the MSP-MS assay by both media conditions. Arrows indicate the location of cleavage sites.

*C*. *neoformans* was cultured in either microbial minimal media (yeast nitrogen base [YNB] pH 5.0) or mammalian tissue-culture media (DMEM pH 7.4), and supernatants from each condition were assayed using a panel of internally quenched (IQ) fluorogenic peptides (Fig 1A, S1 Table for sequences). These substrates were previously developed to detect a broad range of microbial peptidases from diverse peptidase families [40–42]. The speed and flexibility of this assay allowed us to optimize the conditions for peptidase activity and to determine which class-specific inhibitors affect it.

Although peptidase activity was evident under both culture conditions, differential substrate cleavage reflected differences in specificity. Notably, IQ-2 and IQ-6 were cleaved more efficiently by peptidases in YNB media, while proportionally higher activity was observed against IQ-3 and IQ-4 in DMEM media (Fig 1A). These differences suggested that alternate peptidases were active in each culture condition, which was further confirmed by assaying the substrates in the presence of class-specific peptidase inhibitors. This analysis revealed that aspartyl peptidase activity was present in YNB conditioned media while metallopeptidase activity could be detected in DMEM media (S1 Fig). Adjustment of YNB supernatants from pH 5.0 to 7.4 (the pH of DMEM media) yielded only very low levels of any peptidase activity, while lowering the pH of DMEM supernatants from 7.4 to pH 5.0 produced a peptidase activity pattern similar to YNB media (S1 Fig). This result suggests that growth in DMEM media using mammalian cell culture conditions induces peptidases optimized for neutral pH in addition to the acidic pH activities detected after growth in YNB media.

To investigate global peptidase substrate specificity, MSP-MS was conducted on YNB and DMEM supernatants at the optimal pH for activity observed for each condition, pH 5.0 and 7.4, respectively. In the MSP-MS assay, peptide sequencing by LC-MS/MS is used to identify all peptide cleavage products within the 228-member library, revealing peptidase substrate specificity preferences (Fig 1B). The reproducibility of these substrate specificity preferences, and of the cleavage events from which they derive, was confirmed by assessing three technical replicate samples (S2 Fig and S2 Table). Importantly, since there are no modifications to either the N- or C-termini in the peptide library, both exo- and endo-peptidases can be identified in an unbiased manner.

Using MSP-MS we observed that peptidases in YNB media cleaved at 423 total sites, whereas peptidases in DMEM media cleaved at 283 total sites (Fig 1C). Only 107 of these sites were cleaved by enzymes in both samples. This difference in cleavage site preference indicated that peptidase activity and specificity differs between the two culture conditions. Positional analysis of all bonds cleaved within the tetradecapeptides of the MSP-MS library illustrates the proportion of endo- and exo-peptidase activity in each sample (Fig 1D). In YNB supernatants, the most frequently cleaved bond was the carboxyl terminal bond between amino acids thirteen and fourteen, representing 32% of total proteolysis events. In fact, 137 of the 228 peptides had their carboxyl-terminal amino acid cleaved. Moreover, we observed that single amino acids were often sequentially hydrolyzed from the carboxyl termini of substrates until an unfavored residue was reached, consistent with the presence of abundant carboxypeptidase activity. This cleavage preference was not observed for proteases in the DMEM media. These studies indicate that carboxypeptidase activity is more abundant in conditioned media from *C*. *neoformans* cultures grown in YNB. To further illustrate the differences in proteolytic activity between the two conditions, representative examples of peptides cleaved in both samples are shown (Fig 1E).

### Identification of secreted peptidases by proteomic and genetic approaches

To identify which *C*. *neoformans* peptidases may be contributing to the global substrate specificity profile, we conducted a proteomic analysis of secreted proteins. We observed 199 and 131 proteins in YNB and DMEM conditioned media respectively, with 52 proteins common to both conditions (S3 Table). Recently, Geddes and colleagues identified 61 proteins in the secretions of *C*. *neoformans* grown for 16 to 120 hours in minimal media [43], while Campell and coworkers identified 22 secreted proteins after 168 hours growth in RPMI media [29]. In total, 24 of the proteins detected in our study contained predicted secretion signals (SignalP 4.0) [44], 127 were predicted to be non-classically secreted (SecretomeP 2.0) [45], and 17 have been associated with extracellular microvesicles [46]. The remaining proteins identified had no known mechanism of secretion (S3 Fig, S3 Table). Seven of the proteins with predicted signal sequences were peptidases and included members of the aspartyl, metallo and serine peptidase families. Both endopeptidases and carboxypeptidases were identified, consistent with our predictions from analysis of *C*. *neoformans* extracellular proteolytic activity (Fig 1). Five of these enzymes have been detected in studies of the *C*. *neoformans* secretome by other groups; however Prc1 and CNAG_05872 have not been observed previously.

To determine which enzymes are responsible for the proteolytic activity present in *C*. *neoformans* conditioned media, we performed targeted gene deletions on ten candidate secreted peptidases (Table 1, S4 Table). Of the seven aforementioned peptidases with predicted signal sequences that were identified by our secretome proteomics, one was predicted to be GPI-anchored (CNAG_04380) [27,47], and thus excluded from further analysis, as our study was focused on non-cell wall anchored enzymes. One other peptidase could not be mapped unambiguously to a single gene, as three paralogs of this enzyme exist in the *C*. *neoformans* var *grubii* genome [48]. Therefore, all three genes were individually targeted for deletion (*CNAG_00919*, *CNAG_01040* and *CNAG_02966*). Because these genes are unnamed and lack orthologs in *Saccharomyces cerevisiae*, we propose naming them ***C****arbo****x****ypeptidase* ***D 1***, ***2*** and ***3*** (*CXD1-3*), respectively. This resulted in eight genes deleted based on our proteomics results (Table 1, S4 Table). We additionally targeted two secreted peptidases that were not identified here but have been reported in previous proteomics studies [27]. Two independent isolates of each of the ten deletion strains were generated and are indicated in the text and figures by the gene name or CNAG number followed by “-1” or “-2” (S4 Table).

**Table 1:** Peptidase deletion strains generated in this study. Gene names were determined where possible by following the recommended naming guidelines for *C*. *neoformans* [49]. *Nat*^*R*^ is nourseothricin resistance. An asterisk indicates the observation of a phenotype in subsequent mutant characterization studies (S8 and S9 Figs). Evidence for activity in YNB or DMEM conditioned media was determined in subsequent experiments analyzing proteolytic activity in media conditioned by the peptidase deletion strains (Figs 2 and 3, S4 and S5 Figs). Proteins identified in the present study’s secretome proteomics are indicated.

Genotype	Name	Peptidase Type	Proteomics identification	Evidence for activity		Prior secretome identification
				YNB	DMEM	
*CNAG_05973Δ*::*Nat*^*R*^	*SCX1*	Serine carboxypeptidase				[27]
*CNAG_06640Δ*::*Nat*^*R*^	*PRC1*	Serine carboxypeptidase	**+**			
*CNAG_00919Δ*::*Nat*^*R*^	*CXD1*	Carboxypeptidase D	**+**	**+**		[27]
*CNAG_01040Δ*::*Nat*^*R*^	*CXD2*	Carboxypeptidase D	Predicted homolog			[48]
*CNAG_02966Δ*::*Nat*^*R*^	*CXD3*	Carboxypeptidase D	Predicted homolog			[48]
*CNAG_00150Δ*::*Nat*^*R*^	-	Serine endopeptidase				[27]
*CNAG_04625Δ*::*Nat*^*R*^	PRB1*	Serine endopeptidase	**+**			[27]
*CNAG_00581Δ*::*Nat*^*R*^	PEP4*	Aspartyl endopeptidase	**+**			[27]
*CNAG_05872Δ*::*Nat*^*R*^	MAY1*	Aspartyl endopeptidase	**+**	**+**		
*CNAG_04735Δ*::*Nat*^*R*^	*MPR1*	Metallo endopeptidase	**+**		**+**	[27]

Based on our characterization of secreted peptidase activity present in wild type *C*. *neoformans*, we selected deletion strains for in-depth substrate profiling analysis by MSP-MS under either DMEM or YNB culture conditions. Subsequently, by comparing the secreted peptidase activity in conditioned media from the wild type and mutant strains, we were able to correlate extracellular proteolytic activities to specific candidate enzymes.

### DMEM conditioned media contains a metallopeptidase Mpr1 and trypsin-like endopeptidase activity

**Fig 2:**
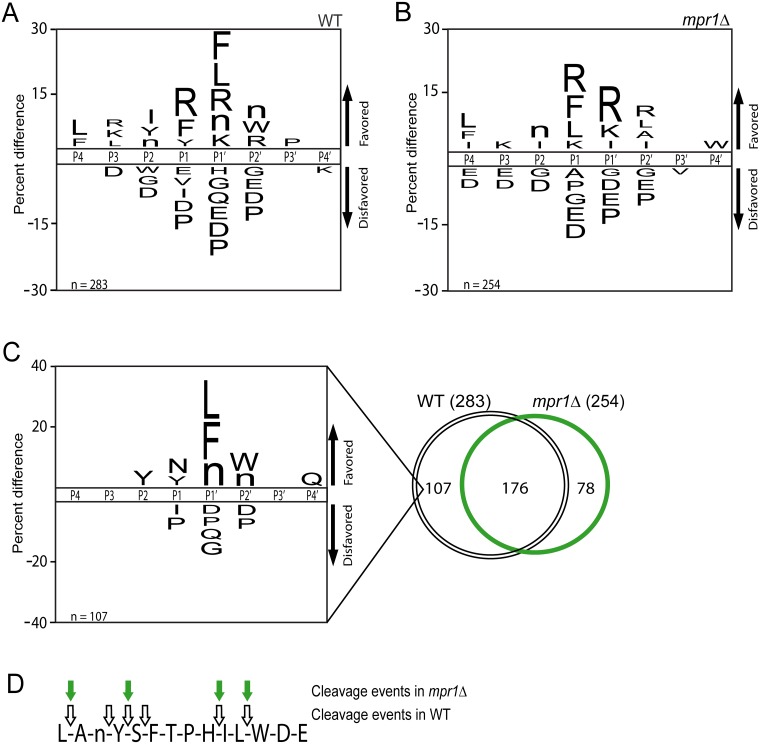
DMEM conditioned media contains a metallopeptidase and trypsin-like endopeptidase activity. (A) The peptidase substrate specificity profile of DMEM media conditioned by wild type. Residues are significantly favored or disfavored as determined by the frequency of detection in substrates versus the frequency of the residue in the peptide library, p < 0.05. (B) Substrate specificity profile of peptidase activity from *mpr1Δ* conditioned media, p < 0.05. (C) Peptidase substrate specificity profile constructed from cleavage events detected in wild type but not *mpr1Δ*, p < 0.05. (D) A representative peptide cleaved by peptidases in both wild type and *mpr1Δ* conditioned media.

To analyze the peptidase substrate specificity of DMEM media conditioned by wild-type cells, we generated a frequency plot from the 283 cleavage events detected by MSP-MS (Fig 2A, S5 Table) [50]. The amino acid preferences are shown for four positions on either side of the cleaved bond (P4-P4'), as the majority of substrate specificity is determined by residues closest to the scissile bond. This analysis revealed that peptidases in DMEM supernatants prefer positively charged residues on either side of the cleaved bond, as well as hydrophobic residues in the P1' position. Negatively charged amino acids are disfavored at the majority of positions, and proline and glycine are both highly disfavored in most positions from P2-P2' (Fig 2A).

To identify the enzymes responsible for this activity, we examined proteolytic activity in peptidase deletion strains. Because DMEM conditioned media contained metallopeptidase activity (S1 Fig) and a single metallopeptidase (Mpr1) was identified by proteomics (Table 1, S3 Table), we began by investigating the contribution of this enzyme to the global specificity profile. Mpr1 had previously been characterized as a secreted factor that is important for *C*. *neoformans* invasion of the CNS [28]. Matched comparison of the substrate specificity profiles obtained from DMEM media conditioned by wild type or *mpr1Δ* cells revealed that Mpr1 deficiency caused a loss of the P1' preference for hydrophobic amino acids seen in wild type (Fig 2A and 2B). However, the selection for positively charged residues on either side of the cleaved bond remained unaltered and the same amino acids were disfavored in most positions.

To further analyze the impact of *MPR1* deletion, a Venn diagram was used to compare the overlap of cleavage events between wild type and *mpr1Δ* (Fig 2C). A majority of cleaved peptides were detected in both samples; however 107 cleavage events were detected in wild type but not media conditioned by *mpr1Δ*. These cleavages, presumed to be absent due to the loss of this enzyme, were used to generate a frequency plot representing the putative specificity of Mpr1 (Fig 2C). A prominent feature of this substrate specificity profile is enrichment for phenylalanine, leucine and norleucine (a replacement for methionine in the MSP-MS library) at the P1' position, a result that is consistent with the specificity of other peptidases predicted to be related to this enzyme (members of the M36 peptidase family) [51]. It is also notable that the P1' site exhibits the greatest degree of selectivity of any position from P4-P4'. To further illustrate the changes in substrate specificity observed in the *mpr1Δ* deletion strain, a representative example of a peptide cleaved by enzymes in both wild type and *mpr1Δ* supernatants is shown (Fig 2D).

An additional activity in DMEM media conditioned by wild type displays a trypsin-like preference for proteolysis between two positively charged residues, indicating the presence of serine peptidase activity [51]. This specificity is particularly evident in the *mpr1Δ* culture media (Fig 2B). Two serine endopeptidases were present in the deletion collection and DMEM conditioned media was analyzed from both strains (*prb1Δ* and *CNAG_00150Δ*). Deletion of either gene did not substantially impact the extracellular peptidase activity profile, suggesting functional redundancy or the existence of additional, unidentified peptidases (S4 Fig). One predicted serine peptidase with a secretion signal, *KEX2*, was identified in a genome search. However, our attempts to delete this gene were unsuccessful, indicating it may be essential for *C*. *neoformans* survival.

In some cases, media conditioned by knockout cells produced additional peptide cleavage sites as compared to wild type conditioned media, despite similar overall peptidase specificity profiles (e.g., S4C Fig). This observation is consistent with the fact that iterative cleavage of an MSP-MS substrate peptide can hinder identification of a given cleavage event due to loss of the cleavage’s reaction product. In this way, the loss of a minor peptidase activity can result in the appearance of new cleavage sites [25].

### An aspartyl endopeptidase May1 and the carboxypeptidase Cxd1 are the major activities in conditioned YNB media

**Fig 3:**
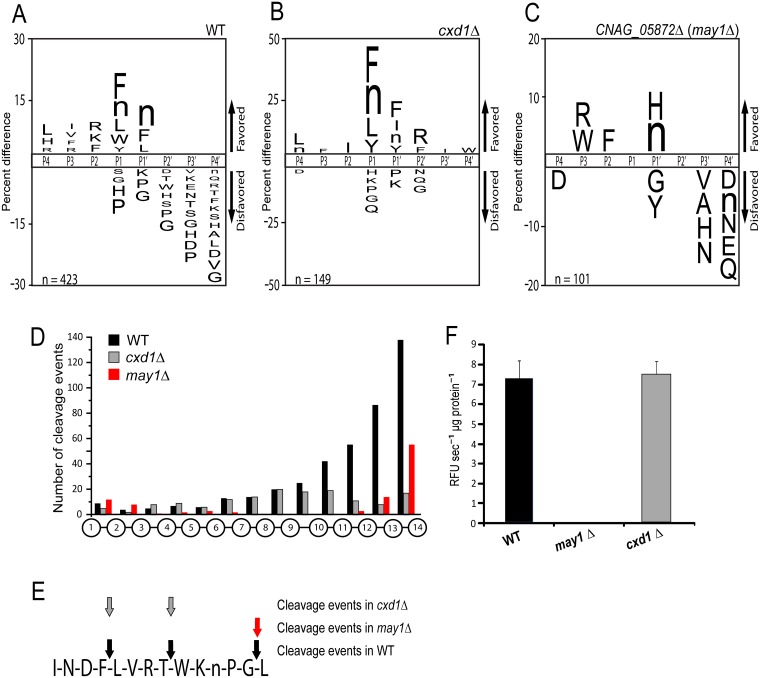
May1 and Cxd1 are the major proteolytic activities in YNB conditioned media. (A) The substrate specificity profile of YNB media conditioned by wild type, p < 0.05. (B) The substrate specificity profile of the carboxypeptidase D deletion strain *cxd1Δ*, p < 0.05. (C) The substrate specificity profile of the aspartyl peptidase deletion strain *may1Δ*, p < 0.05. (D) Positional profiling of all bonds cleaved within the tetradecapeptides of the MSP-MS library. (E) A representative example of a peptide in the MSP-MS library cleaved by wild type and both deletion strains. (F) Deletion of May1 abolishes endopeptidase activity against IQ-2. Columns represent mean ± S.D.

A substrate specificity profile constructed from the 423 cleavages observed in YNB media conditioned by wild-type cells indicated a preference for hydrolysis between hydrophobic residues, while positively charged residues, proline and glycine are disfavored (Fig 3A). From positional analysis of these cleavage sites, we identified carboxypeptidase activity as the dominant proteolytic activity in this media (Fig 1D). Since carboxypeptidases cleave the carboxyl-terminal bond, no enrichment of amino acids is evident in the P2' to P4' positions in these substrates (Fig 3A).

To determine whether any of the three carboxypeptidase D paralogs we identified in our proteomics analysis were responsible for the observed carboxypeptidase activity, the gene for each enzyme was deleted and conditioned media from the resulting mutant strains *(cxd1Δ*, *cxd2Δ* and *cxd3Δ)* was profiled by MSP-MS. After comparison of the specificity profiles, it was clear that the carboxypeptidase specificity was most dependent on Cxd1. In media conditioned by this deletion strain only 17 primary carboxyl-termini were cleaved, as compared to 137 in wild type (Fig 3B, 3D and 3E). By comparison, 134 and 123 primary carboxyl-termini were cleaved in media conditioned by *cxd2Δ* and *cxd3Δ*, respectively, suggesting that these enzymes do not contribute substantially to the extracellular carboxypeptidase activity (S5 Fig, S5 Table). As anticipated, endopeptidase cleavages were not affected in any of the three carboxypeptidase deletion strains (Fig 3B and 3D, S5 Fig).

Fluorogenic assays demonstrated aspartyl endopeptidase activity in wild type YNB supernatants (S1 Fig). To assign activity to the candidate aspartyl peptidases, conditioned YNB media was profiled from the two aspartyl peptidase deletion strains listed in Table 1 (*CNAG_05872Δ* and *pep4Δ)*. Proteolytic activity remained unchanged relative to wild type in the *pep4Δ* strain (S5 Fig). In contrast, *CNAG_05872Δ* conditioned media exhibited a near-total loss of endopeptidase cleavage events as well as substantially decreased carboxypeptidase activity as evidenced by proteolysis of only 55 primary carboxyl termini (Fig 3C–3E). This result suggests that CNAG_05872 is the dominant endopeptidase under these culture conditions. This finding is consistent with fluorogenic assays, where deletion of *CNAG_05872* led to a loss of endopeptidase activity in conditioned YNB media, while all other strains exhibited activity levels similar to wild type (Fig 3F, S6 Fig). As the putative dominant endopeptidase, we propose renaming *CNAG_05872* to ***M****ajor* ***A****spart****y****l peptidase 1* (*MAY1*).

### May1 is a pepsin-like aspartyl peptidase, with optimal expression and activity at acidic pH

**Fig 4:**
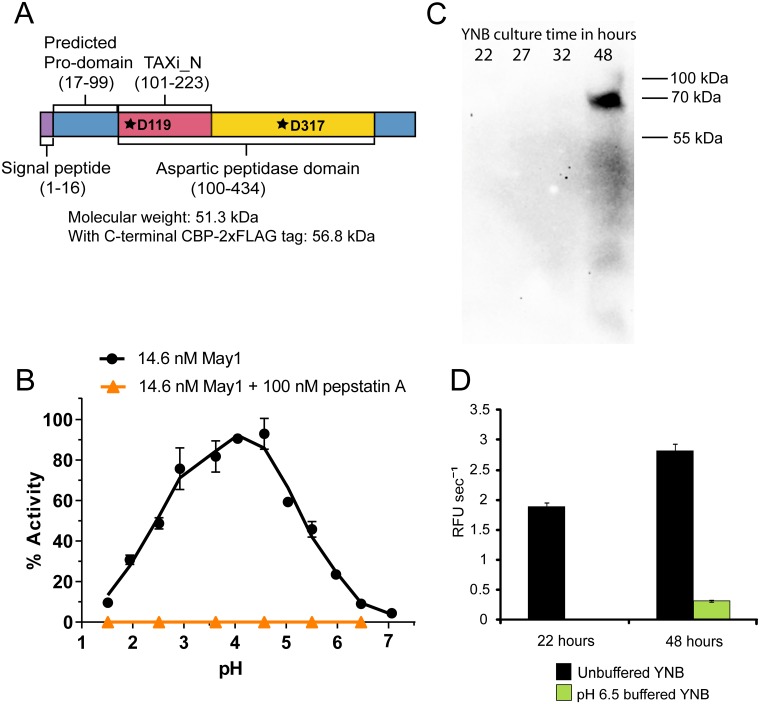
May1 is a member of the pepsin-like aspartyl peptidase family with optimal expression and activity at acidic pH. (A) The domain architecture of May1. The catalytic aspartic acids are indicated by stars. (B) pH titration of May1 activity using IQ-2. Averages and standard deviation (S.D.) of triplicates are shown. (C) Immunoblot detection of May1 tagged with a CBP-2xFLAG tag in supernatants after culturing in YNB for different lengths of time. (D) Activity against IQ-2 in conditioned media from wild type *C*. *neoformans* grown in unbuffered or pH 6.5 buffered YNB. All samples were adjusted to pH 4.5 prior to being assayed. Columns represent mean ± S.D.

Due to its substantial contribution to peptidase activity in YNB supernatants, we performed an in-depth biochemical characterization of May1. This enzyme consists of a 16 residue secretory signal (SignalP 4.0) [44] followed by an 82 residue prodomain (Fig 4A). The prodomain is positively charged (pI = 9.97), which likely facilitates interaction with the negatively charged catalytic domain (pI = 4.03) at neutral or slightly acidic pH [52]. The pepsin-like aspartyl peptidase domain includes residues 100–434 and is expected to auto-activate in acidic environments, causing release of the pro-domain. The N-terminal region (position 101–223) is also an N-terminal xylanase inhibitor domain, TAXi_N [53]. Homology between xylanase inhibitors and fungal aspartyl peptidases has been noted previously and likely indicates an evolutionary relationship [54].

May1 readily cleaves IQ-2 between phenylalanine and leucine (S1 Table, S6 Fig), which allowed us to use fluorogenic assays to monitor enrichment of this enzyme from YNB supernatants and investigate the impact of pH on its activity. Ion exchange chromatography was used to enrich May1 from conditioned YNB media, resulting in a 292 nM peptidase stock solution. May1 was diluted from this stock into buffers ranging from pH 1.5 to 7.0 and activity against IQ-2 was tested. Optimal activity was observed between pH 3.5–4.5, a range that is consistent with other members of the aspartyl peptidase family of enzymes (Fig 4B) [55]. The aspartyl peptidase antagonist pepstatin A fully inhibited proteolysis of IQ-2 in this assay, providing further verification that May1 is the predominant endopeptidase activity under these conditions.

To investigate the time- and growth-dependent secretion of May1, we added a CBP-2xFLAG tag to the carboxyl-terminus through homologous recombination. By monitoring activity using IQ-2, we confirmed that the addition of this tag did not diminish May1 activity in YNB conditioned media. Interestingly, although recombinant May1 activity could be detected in the culture supernatant after overnight growth, it could not be detected by immunoblot even after three days of growth. We hypothesized that the enzyme rapidly hydrolyses the C-terminal tag; therefore pepstatin A was added to the culture to inhibit this processing. This inhibitor also prevented activation of pro-May1 to mature May1, resulting in the observation of only the pro form of recombinant May1. Under these conditions the protein was detected by immunoblot after 48 hours of growth (Fig 4C). The apparent molecular weight of pro-May1 was approximately 13 kDa greater than the predicted 56.8 kDa for the tagged enzyme, suggesting that this protein could contain post-translational modifications. When *C*. *neoformans* was cultured in YNB media buffered to pH 6.5, the May1 activity detectable in supernatants using IQ-2 was approximately 10-fold lower than in unbuffered YNB, and no signal could be seen by immunoblot after 48 hours of growth (Fig 4D). This result suggests that low extracellular pH could stimulate May1 secretion and activation.

### May1 activity is required for wild-type saturation density in YNB cultures

**Fig 5:**
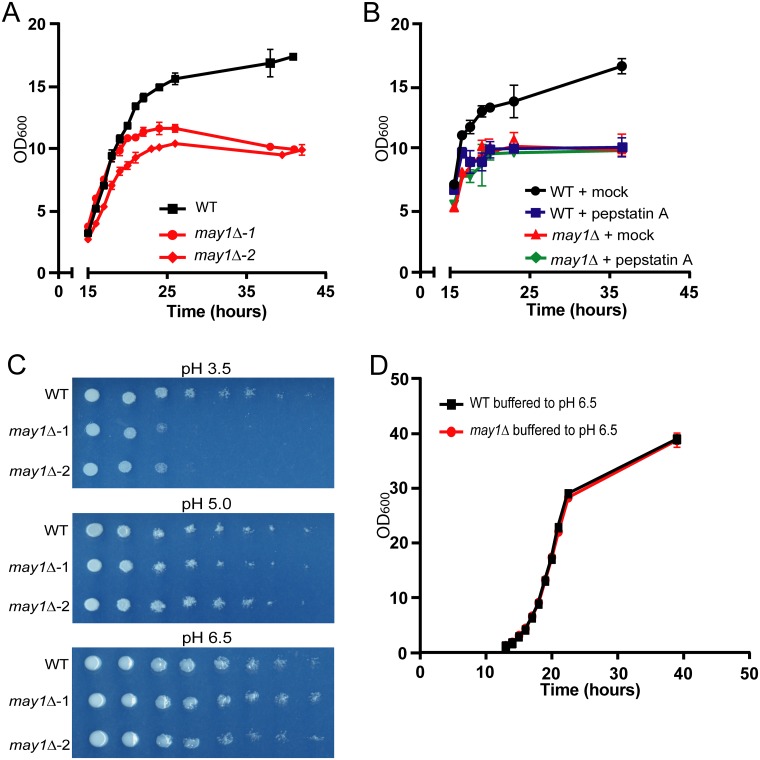
May1 activity is required for wild-type growth at acidic pH. (A) Culture density was recorded over time by measuring OD_600_ of cultures grown in YNB. The average density and S.D. of triplicates are shown. (B) Culture density of wild-type *C*. *neoformans* grown in YNB containing 2 μM pepstatin A was recorded. Mock indicates DMSO treatment. The average and S.D. of cultures grown in triplicate are shown. (C) Mutant characterization assays were conducted using YNB agar plates. Cultures were adjusted to an OD_600_ of 5 and then spotted in 10-fold serial dilutions on plates. (D) Full rescue of *may1Δ* saturation density in YNB was observed after buffering to pH 6.5. Average density and S.D. of triplicates are shown.

We observed that *may1Δ* strains grown in YNB had a lower cell density at saturation than wild type or any of the other nine peptidase deletion strains (Fig 5A, S7 Fig, S6 Table). Higher cell density could not be achieved by the *may1Δ* strains even after culturing for 96 hours. These data suggested that *may1Δ* strains were not merely slow to replicate but were incapable of growth at high density. In fact, during the exponential growth phase, *may1Δ* strains had an average doubling time of 2.36 hours, which was on par with the other nine peptidase deletion strains. However, all other deletion strains exhibited wild-type saturation densities (S6 Table).

Studies of deletion strains did not clarify whether May1 activity, as opposed to simply the presence of the May1 protein, was required to reach a saturation density equivalent to wild type. Therefore, we assessed growth of wild type *C*. *neoformans* in the presence and absence of pepstatin A. Treatment of wild type cultures with this aspartyl peptidase inhibitor resulted in a saturation density defect equivalent to that observed for the *MAY1* deletion strains (Fig 5B). Importantly, the inhibitor had no effect on *may1Δ* cells, suggesting that the growth defect observed in wild-type cells treated with pepstatin A was mediated through inhibition of May1.

### Mutant characterization assays reveal a *may1Δ* growth defect at low pH

Plating assays on various stress conditions were conducted with two independent isolates of each of the ten peptidase deletion strains (S8 and S9 Figs). After 48 hours *may1Δ* strains exhibited a growth defect at pH 3.5 but not at pH 5.0 or 6.5 (Fig 5C). Longer growth periods at pH 3.5 did not allow *may1Δ* colonies to overcome this sensitivity and after three days of growth, it became apparent that *may1Δ* colonies also had a slight defect at pH 5.0 but not pH 6.5 (S8 Fig). None of the other peptidase deletion strains displayed sensitivity to acidic pH, although *pep4Δ* was sensitive to hydrogen peroxide and SDS, and *prb1Δ* had a slight sensitivity to hydrogen peroxide (S8 and S9 Figs). Based on this result, we hypothesized that the inability of *may1Δ* strains to reach wild-type saturation densities in YNB was a result of acidification of the media and would be rescued by buffering the media to pH 6.5. These culture conditions fully rescued the saturation density of *may1Δ* (Fig 5D). Surprisingly, it also allowed the final saturation densities of both wild type and *may1Δ* cultures to approximately double, revealing that low pH is a condition limiting growth even for wild-type *C*. *neoformans*.

We also assessed melanization, an established virulence factor, for each of the ten peptidase deletion strains. Because melanin production occurs extracellularly, we hypothesized that this process could be influenced by secreted peptidase activity. Only the serine endopeptidase deletion strain *prb1Δ* exhibited a hypomelanization phenotype (S9 Fig).

### A screen of an aspartyl peptidase inhibitor library yields compounds antagonistic to May1

While pepstatin A inhibits May1 with an IC_50_ of 1.4 nM, it is a broad acting antagonist of many members of the aspartyl class of peptidases, thereby limiting its utility. To determine whether additional inhibitors targeting May1 could be obtained, we conducted an *in vitro* screen using knowledge of May1 substrate specificity derived from MSP-MS analysis. We screened a panel of 21 peptidomimetic molecules with similarities to May1 substrate preferences but with a non-cleavable bond between the P1 and P1' position (S7 Table). Compounds 1 to 11 are linear peptidomimetics, while compounds 12 through 21 are macrocycles (S7 Table) [56–59]. We also screened ten HIV protease inhibitors because some of these molecules have been reported to inhibit *C*. *neoformans* peptidase activity [60,61].

**Fig 6:**
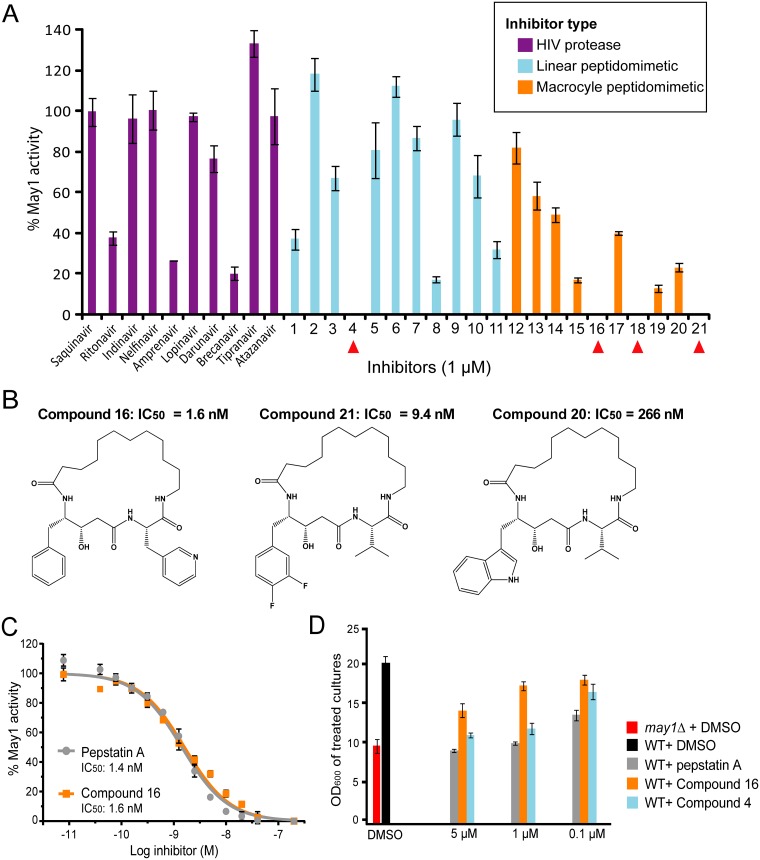
A screen of aspartyl peptidase inhibitors uncovers compounds antagonistic to May1. (A) Three groups of compounds were screened for inhibition of May1 activity using IQ-2. Compounds completely inhibiting May1 at 1 μM are denoted with red triangles. Averages and S.D. of triplicates are shown. (B) The structures of three macrocyclic compounds screened for inhibition of May1. (C) The IC_50_ for the most potent May1 inhibitor (compound 16) was found to be 1.6 nM, while peptstatin A had an IC_50_ of 1.4 nM. The average and S.D. of measurements in triplicate are shown. (D) Density at saturation (after 48 hours of growth) is shown for YNB cultures of wild type *C*. *neoformans* treated with May1 inhibitors. Average values and S.D. of triplicates are shown.

May1 was incubated with 100 μM, 10 μM and 1 μM of each inhibitor and activity was detected using IQ-2 (Fig 6A, S10 Fig). IC_50_ values were then calculated for the most potent compounds. The best inhibition by an HIV protease inhibitor was observed with Brecanavir, which reduced activity by 80% at 1 μM and had an IC_50_ of approximately 352 nM (S10 Fig). Among the peptidomimetic molecules, the macrocycles were the most potent, with the best compounds (15 to 21) containing P2 –P3' tethered side chains, statines in P1 and an α-amino acid in P2' (Fig 6, S7 Table). Compounds 16, 21, and 18 all exhibited nanomolar IC_50_ values of 1.6 nM, 9.4 nM and 41 nM, respectively (S10 Fig). Among the linear peptidomimetic inhibitors, those with a phenylstatine or hydroxyethylamine scissile bond isoster (compounds 4, 7, 8 and 11) were superior to compounds with a reduced bond (1, 2, 5, 6 and 9) or a homo-amide (2). Compound 4 was the most potent May1 antagonist out of this group of inhibitors, with an IC_50_ of 3.1 nM (S10 Fig).

From analysis of the four most effective inhibitors identified in our screen (compounds 4, 16, 18 and 21), it is clear that a phenylalanine side chain, either unsubstituted (4 and 16) or with a small substituent (18 and 21), is preferred in P1 while a bulkier P1 side chain leads to decreased potency, for example compounds 17, 19 and 20. These results match the P1 substrate preference for phenylalanine that we had predicted for May1 and fit our expectations that bulkier residues such as tryptophan are not well tolerated in this position (Figs 3 and 6B).

Next, we selected the two best *in vitro* hits to test their potency in culture relative to pepstatin A by measuring inhibition of May1 and restriction of culture growth using fluorogenic assays and OD_600_ respectively. Wild-type *C*. *neoformans* was grown in YNB treated with 5 μM, 1 μM or 0.1 μM of compound 4, 16 or pepstatin A and the culture density and May1 activity were measured at saturation. While compound 16 exhibited an *in vitro* IC_50_ comparable to pepstatin A, it was not as effective at inhibiting May1 activity or restricting culture growth (Fig 6D, S11 Fig). Curiously, despite having an *in vitro* IC_50_ approximately twice that of compound 16, compound 4 was better at inhibiting culture growth. None of the three compounds affected the culture density of a *may1Δ* strain, consistent with the idea that May1 is the compounds’ relevant target in this context (S11 Fig). These results demonstrate that May1 can be targeted by small molecule inhibitors and provide a discovery framework for further inhibitor development. However, additional medicinal chemistry efforts are necessary for *in vivo* applications. Therefore, subsequent studies investigating the role of May1 in virulence were carried out using deletion strains of this enzyme.

### *May1* is required for virulence

**Fig 7:**
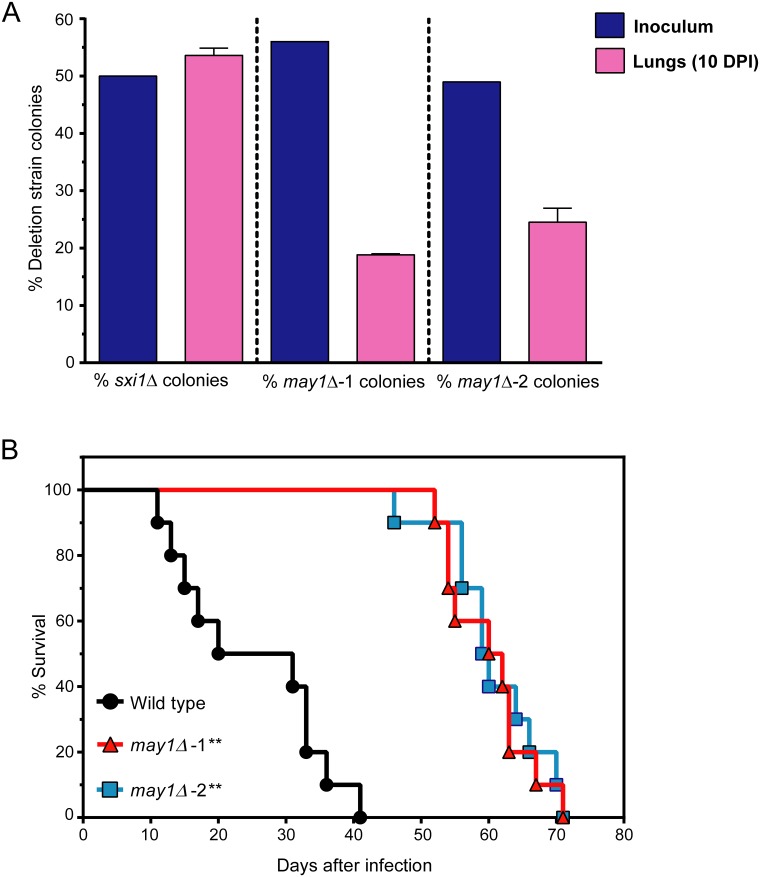
May1 is required for virulence in a mouse inhalation model of infection. (A) Three mice per group were infected with a 1:1 ratio of wild type to mutant cells using the mutant strains *may1Δ*-1, *may1Δ*-2 or *sxi1Δ*. The 1:1 ratio of wild type to mutant strain was confirmed by plating the inoculums on plates containing nourseothricin as a selection agent. Ten days after infection, lungs were harvested and plated to determine the ratio of wild type to deletion strain. Average values and S.D. are shown. DPI: days post infection. (B) Ten mice per group were infected with wild type, *may1Δ*-1 or *may1Δ*-2 cells. Significance was determined by a log-rank test, ** indicates p < 10^−5^.

Because *may1Δ* strains exhibit phenotypes in both peptidase activity assays and growth at low pH, we examined the role of this protein in virulence using an established mouse inhalation model of Cryptococcal infection [81]. Wild-type cells were mixed with an equivalent number of *may1Δ* cells and used to infect mice intranasally (Fig 7A). These experiments were conducted using two independent isolates of *may1Δ* as well as a negative control known not to affect fungal replication in this assay (*sxi1Δ*) [63]. In each isolate, genes neighboring *may1Δ* were tested by RT-qPCR to confirm expression (S12 Fig). Ten days after infection, mouse lungs were harvested and plated for colony forming units (CFUs), at which point *may1Δ* strains contributed only 22 ± 3% of the colonies recovered from the lungs, a substantial decrease from the approximately 50% present upon infection. This result reveals that *may1Δ* cells have a growth defect within a mammalian host because the ratio between deletion strain and wild type cells was reduced after host infection.

Given the defect in *may1Δ* cell accumulation in the lung, we evaluated the role of May1 during *Cryptococcus* interaction with macrophages. Opsonized wild type and *may1Δ* strains were phagocytosed with equal efficiency by bone marrow-derived macrophages (S13A Fig). We next tested the ability of *may1Δ* cells to accumulate within macrophages after phagocytosis, since the phagolysosome environment may represent a low-pH setting in which May1 is active. Indeed, cells lacking May1 accumulated significantly more slowly within macrophages than did wild type cells (S13B Fig), consistent with a role for May1 within host cells.

The defect in accumulation of *may1Δ* cells during intranasal infection and within macrophages suggested that these strains would be attenuated for virulence. We directly investigated the virulence of *may1Δ* strains by performing monotypic infections [62]. Ten mice per group were infected intranasally with wild type, *may1Δ*-1 or *may1Δ*-2 cells (Fig 7B). Loss of May1 caused significant attenuation of virulence, with mice infected by *may1Δ*-1 or *may1Δ*-2 exhibiting a mean survival time of 60.1 and 60.7 days respectively, whereas those infected by wild type had a mean survival time of 25 days. The results from these *in vivo* experiments indicate an important role for May1 during mammalian infection.

## Discussion

In this work, we investigated secreted proteolytic activity in *C*. *neoformans* var. *grubii* culture media using an unbiased approach that can detect both endo- and exo-peptidase activity. In combination with proteomic methods and single gene deletion approaches, this strategy allowed us to characterize peptidase activity from a global perspective as well as interrogate the roles of individual enzymes in the *C*. *neoformans* secretome. By comparing the overlap in peptidase activity between wild type and these deletion strains, we were able to identify and define the substrate specificities of a carboxy, aspartyl and metallopeptidase which contribute substantially to the total peptidase activity profile. Additionally, we delineated the substrate specificity of an unidentified trypsin-like peptidase activity, an intriguing result given previous reports implicating secreted serine peptidases in *C*. *neoformans* pathogenicity [31,34].

Deletion of some peptidase genes, such as the predicted carboxypeptidase D genes *CXD2* and *CXD3*, caused no significant change in secreted proteolytic activity or cellular phenotype. Instead, it appears that a third carboxypeptidase D paralog *CXD1* is responsible for the majority of exopeptidase activity under these conditions. The broad specificity of Cxd1 suggests that one possible role for this enzyme could be in nutrient acquisition by providing *C*. *neoformans* with free amino acids from extracellular protein sources [17,18,64].

The serine peptidase deletion strain *prb1Δ* also had a minimal effect on total secreted peptidase activity; however, a phenotype of reduced melanin production was evident, indicating function under these conditions (S9 Fig). One possibility is that this gene encodes an enzyme with very strict substrate specificity, thus its deletion did not have a substantial impact on total extracellular peptidase activity as measured by the MSP-MS assay (S4 Fig).

Through the application of our global profiling approach to different culture conditions, we were able to demonstrate that the landscape of secreted peptidase activity shifts in response to alterations in environment. This result raises the possibility that changes in extracellular proteolytic activity could be relevant for adaptation. For example, we detected the activity of the metallopeptidase Mpr1 only after growth under neutral pH conditions, whereas we find that May1 is optimally active under acidic conditions. Thus, these enzymes may function in different settings within the host or within other environments encountered by *C*. *neoformans*.

Through proteolytic profiling and mutant characterization assays, we identified the aspartyl peptidase May1 as the dominant endopeptidase at low pH and found that its activity is required for tolerance to acidic environments. The strongest determinant of May1 substrate specificity was shown to be a preference for cleavage between hydrophobic residues, in particular phenylalanine, leucine and norleucine (Fig 3A). Based on these results, we screened a focused panel of aspartyl peptidase inhibitors with similarity to the P1-P1' substrate specificity of May1. Several of these compounds had IC_50_ values in the nanomolar range, whereas the HIV protease inhibitors had relatively poor affinity for May1. Previous reports have shown that some HIV protease inhibitors reduce secreted aspartyl peptidase activity produced by *C*. *neoformans* [60,61]. The concentrations of inhibitors required to achieve statistically significant inhibition in previous studies were much higher than those used in the experiments reported here although the trends for inhibitor potency match our results [60]. Therefore, it is possible that the inhibition of *C*. *neoformans* aspartyl peptidase activity seen in previous publications could be explained by the inhibition of May1.

We found that strains lacking May1 are attenuated in a competition infection assay, a macrophage accumulation assay and a monotypic infection assay. In microbial culture it is likely that May1 cleaves one or more secreted or cell wall-bound fungal proteins to facilitate low pH tolerance. However, it is possible that during an infection May1 cleaves host proteins and either or both of these proteolytic events impacts virulence. An additional important consideration for defining the role of May1 in *C*. *neoformans* pathogenicity is the cleavage context within the host. Our pH titration determined that May1 has very low levels of activity above pH 6.5; however few environments of lower pH than this exist within the mammalian host. Therefore, it is possible that residual May1 activity at neutral pH is important, or alternatively that May1 could be relevant for survival in acidic host environments such as dendritic cell or macrophage phagolysosomes, which exhibit a pH of ~5.0 in the context of Cryptococcal phagocytosis [65,66]. A third possibility is that a combination of these factors contributes to the attenuated virulence of *may1Δ* strains.

We have identified orthologs of *MAY1* in many other basidiomycetes including the opportunistic pathogens *Trichosporan asahii* and *Cryptococcus gattii*, the latter of which is capable of infecting immunocompetent individuals [67] [48]. Many pathogenic ascomycetes also contain *MAY1* orthologs, including *Histoplasma capsulatum*, *Coccidioides immitis* and *Aspergillus* species, although the sequence identity is low [48]. None of the *MAY1* orthologs in basidiomycetes has been well studied and only one ortholog in an ascomycete has been examined. This enzyme, from *A*. *fumigatus*, encodes a protein secreted during infection of the virulence model *Galleria mellonella* [68]. The hypovirulent phenotype observed in *C*. *neoformans may1Δ* strains and the identification of May1 orthologs in other fungal pathogens raises the possibility that this peptidase family displays a conserved virulence function and suggests that the roles of these orthologs are important to investigate.

Small molecule drug development requires a thorough understanding of the target enzyme as well as the surrounding peptidase milieu [69–73]. The results described in this report lay the groundwork for investigating the functions of *C*. *neoformans* secreted peptidases and the use of inhibitors to modulate their activity. The substrates and inhibitors presented here may also be of value for interrogating related fungal peptidases. Furthermore, our approach to investigating secreted peptidases through integration of activity profiling, proteomics, and genomics strategies is broadly applicable to other genetically tractable pathogens.

## Materials and Methods

### Ethics statement

Studies in mice were carried out according to the recommendations in the Guide for the Care and Use of Laboratory Animals of the National Institutes of Health. All protocols were reviewed and approved by the Institutional Animal Care and Use Committee, University of California, San Francisco, approval number AN091509-02C. During infections, mice were anesthetized by an intraperitoneal injection of ketamine (75 mg/kg) and dexmedetomidine (0.5 mg/kg), which was reversed by an intraperitoneal injection of atipamezole (1.5 mg/kg). Mice were sacrificed in accordance with protocol guidelines by CO_2_ inhalation and cervical spine dislocation.

### Peptide-based detection of peptidase activity and characterization of substrate specificity

#### Fluorogenic peptide assays

Assays were conducted at room temperature on a Biotek Synergy H4 plate reader in a 50 μl volume using 96-well round bottom, polystyrene plates (Corning) with λ_ex_ 328 nm λ_em_ 393 nm unless otherwise stated. Substrates were each 7 or 8 amino acids with 7-methoxycoumarin-4-acetic acid or 7-methoxycoumarin-4-yl-acetyl-L-lysine on the amino terminus, and at the carboxyl terminus 4-dinitrophenyl-L-lysine or 4-dinitrophenyl bound directly to the carboxyl terminus as indicated (For sequences see S1 Table). Substrates were assayed at a 10 μM final concentration from DMSO stocks. Biotek Gen5 software was used to calculate initial velocities in relative fluorescent units per second (RFU/sec) from 20 points over the linear portion of each assay. To assay activity, YNB conditioned media was adjusted to pH 4.5 using 100 mM MES, 100 mM NaCl buffer, pH 6.5 at a final concentration of 26 mM MES, 26 mM NaCl, unless otherwise stated. Conditioned DMEM media was first buffer exchanged into PBS using a centrifugal filter unit with a 3 kDa cutoff (Millipore) before use. The peptidase inhibitors pepstatin A, 1-10-phenanthroline, AEBSF and E64 were dissolved in DMSO and were obtained from Sigma-Aldrich.

#### Multiplex substrate profiling by mass spectrometry

Full methods are available elsewhere [39]. Minor modifications to the published method are as follows: The library contained 104 additional tetradecapeptides designed using the same algorithm as published for a total of 228 synthetic peptides. The library was split into two pools of 114 peptides to optimize detection by LC-MS/MS and 500 nM of each peptide was present in the assay. YNB supernatants (32 hour cultures) were adjusted to pH 5 and diluted 1:2 in fresh YNB prior to assaying by MSP-MS, whereas DMEM supernatants (32 hour cultures) were buffer exchanged into PBS and used undiluted in the assay. The assay was conducted at room temperature and samples were removed at the time points defined [39].

Mass spectrometry was conducted on either the LTQ Orbitrap XL or an LTQ FT machine as described [39]. The full length sequences of all substrates were then deduced by comparison to the intact peptides found in the library using the Protein Prospector program v5.10.15 (UCSF), and an excel format of the results was generated using the Extractor program (UCSF) [39,74]. The frequency with which each amino acid was detected in the P4 to P4' positions was illustrated using iceLogo software [50]. All possible P4 to P4' sequences in the 228-member library were used as the reference dataset (S5 Table).

### Proteomics

Conditioned media was prepared from wild type *C*. *neoformans* cultured in YNB (32 hours) or DMEM (48 hours) as described below and concentrated using Millipore centrifugation filters, (3 kDa molecular weight cutoff). Trypsin digestion was conducted as previously described using a 1/40 mass ratio of trypsin/protein [39]. Peptides were recovered and desalted using C18 tips (Rainin). Peptide identification was conducted as previously described using the LTQ-Orbitrap XL mass spectrometer (Thermo) [25]. To identify proteins, searches were carried out against the Uniprot database (downloaded March 21, 2012), with *Cryptococcus* species entered as the taxonomy. This database was concatenated with a fully randomized set of proteins for determination of false-identification rate. Peptides were matched with up to 2 missed trypsin cleavages, carbamidomethylated cysteines as a fixed modification and oxidation of methionine, N-terminal methionine loss with or without acetylation, N-terminal acetylation or oxidation and pyroglutamate from glutamine at the N-terminus as variable modifications. Tolerance for mass accuracy was 20 ppm for parent and 0.8 Da for fragment errors.

For protein identification from the database search, the Protein Prospector settings were: 15 for the minimum protein score and 10 for the minimum peptide score. The maximum expectation value for proteins was set at 0.009 and for peptides it was 0.05. At the time of this study, the Uniprot database did not contain annotated *C*. *neoformans* var *grubii* genes, thus protein matches were identified within other *C*. *neoformans* serotypes and the var *grubii* orthologs were identified by searching the H99 genome either manually or through BLASTp searches using the NCBInr database (http://blast.ncbi.nlm.nih.gov/blast/Blast.cgi).

SignalP version 4.0 was used to predict secretion signals, while SecretomeP version 2.0 was used to predict non-classical secretion pathways [44,45]. Data are reported in S3 Table.

Identification of May1 orthologs was conducted by searching for *CNAG_05872* in FungiDB (www.fungidb.org) [48]. The functional domains of May1 were annotated using BLASTp. Isoelectric point and molecular weights were determined using ExPASy (http://www.expasy.org/) [52].

### Yeast genetics

#### Yeast strains

*C*. *neoformans* genes were defined by Broad Institute (Cambridge, MA) annotations of the var. *grubii* H99 genome (http://www.broadinstitute.org/annotation/genome/cryptococcus_neoformans/MultiHome.html), where each gene is named numerically as “CNAG_#” [75]. All *C*. *neoformans* strains used in this study were derived from strain H99 using standard procedures [62] (S4 Table). If unpublished, names for *C*. *neoformans* peptidases were assigned following the guidelines established in Inglis *et al*. [49].

#### Preparation of conditioned media

Yeast cultures were grown in either YNB (1.5 g/L yeast nitrogen base, 5 g/L ammonium sulfate, 2% glucose) or in Dulbecco’s Modified Eagle Medium (DMEM) without phenol red (4.5 g/L glucose, 0.584 g/L L-glutamine, 3.7 g/L NaHCO_3_). YNB media is unbuffered and has a starting pH of 5.0, acidifying to a final pH between 1.5–2.0 in saturated cultures, while DMEM is buffered to pH 7.4. For YNB conditioned media, a single yeast colony was inoculated into 100 ml YNB and grown with shaking for a defined duration at 30°C (32 hours unless otherwise noted). The cultures were then centrifuged; the supernatant was filtered (0.45 μm), flash frozen and stored at -20°C. For DMEM conditioned media, 90 ODs of log-phase *C*. *neoformans* cells grown in YNB (the equivalent of 90 ml of a culture at an optical density at 600 nm (OD_600_) of 1) were centrifuged, inoculated into mammalian cell culture dishes containing 25 ml DMEM and maintained in a tissue culture incubator at 37°C with 5% CO_2_. After 32 hours (unless otherwise noted), the media was harvested as described above. Because *C*. *neoformans* responds to light, strains were grown in darkness [76].

#### Mutant characterization assays

Overnight YNB cultures were adjusted to an OD_600_ of 5 and 3 μL spots of 10-fold dilutions were spotted onto 2% YNB agar plates. Growth at 37°C was measured as well as tolerance to low pH, high pH, NaCl, hydrogen peroxide, sorbital, caffeine and SDS were measured through inclusion of 25 mM succinic acid, 25 mM MES pH 6.5, 0.75 M NaCl, 0.5 mM peroxide, 1 M sorbitol, 26 mM caffeine and 0.02% SDS respectively. Melanization using L-DOPA containing plates was assayed as previously described [77]. Melanization and all plate assays apart from growth at 37°C were conducted at 30°C. Doubling times were calculated using http://doubling-time.com/compute.php [78].

#### RNA isolation and RT-qPCR

*C*. *neoformans* cultures were grown at 30°C in YNB medium until log phase (OD_600_ = 1.0) or stationary phase (32 hr), then harvested by centrifugation and snap frozen. Cells were lyophilized overnight, after which RNA was extracted using TRIzol (Invitrogen). Total RNA was treated with DNaseI (Roche) and reverse transcribed by addition of SuperScript III (Invitrogen), using oligo-dT20N (38 ng/μl) and random 9-mers (10 ng/μl) as primers. The cDNA was treated with RNaseH (New England Biolabs) and assessed by qPCR using a CFX96 Real-Time system (Bio-Rad).

### May1 characterization and enzymatic assays

#### Immunoblot

Samples were collected at the designated time points from liquid YNB cultures and OD_600_ determined. 42 ODs (the equivalent of 42 ml of a culture at an OD_600_ of 1) were then centrifuged and the supernatant removed and frozen. The samples were lyophilized and then dissolved in 0.17 mM Tris base pH 8 and 1X SDS loading dye containing tris(2-carboxyethyl)phosphine. After boiling for 15 minutes the samples were loaded onto a 4–12% Bis-Tris gel and run using MES buffer (Life Technologies). Gels were transferred to nitrocellulose membranes using the iBlot dry transfer system (Life Technologies) and blocked in 2% BSA. The monoclonal mouse anti-flag primary antibody (Sigma-Aldrich) diluted 1:2,000 in 2% BSA was used followed by HRP conjugated goat anti-mouse secondary antibody (Thermo Scientific) diluted 1:10,000 in 2% BSA. The Luminata Forte Western HRP substrate was used (EMD Millipore) and the blot imaged using a BioRad ChemiDoc imager.

#### Determination of May1 cleavage site within IQ-2

Matrix assisted laser desorption ionization-time of flight (MALDI-TOF) analysis was conducted to identify the site of May1 cleavage within IQ-2. 100 μM IQ-2 was digested in a 50 μl assay with 14.6 nM May1 in 100 mM MES, 100 mM NaCl pH 4.5. 10 μl samples were collected at the start of the reaction and after 24 hours. Peptides were recovered and desalted using Rainin C18 tips, lyphophilized, and redissolved in 5 μl 0.1% formic acid. 0.5 μl of sample was combined with 0.5 μl matrix and analyzed by MALDI-TOF (Shimadzu Biotech Axima Performance).

#### May1 enrichment

To concentrate secreted May1, YNB conditioned media was prepared as described above from 32-hour cultures of wild-type *C*. *neoformans*. The media was then diluted 2.7-fold into buffer A (50 mM Tris base pH 8), chosen to increase the pH in order to limit May1 autoproteolysis, dilute salts in the media (final conductivity ~6%), and confer a negative charge to the peptidase domain. The media was then loaded onto a 1 ml HiTrap DEAE fast flow column (GE Healthcare) using a fast protein liquid chromatography system with a flow rate of 1.5 ml/min. May1 was eluted using a 30 minute linear gradient of 0–100% buffer B (50 mM Tris base, 1 M NaCl, pH 8). Active fractions were determined by measuring activity using the substrate IQ-2. They were then combined and approximate May1 concentration determined by active-site titration.

#### Active site titration, Km and IC_50_ calculations

For the following experiments the plate reader conditions were as described for fluorogenic assays and the substrate used was IQ-2 at 10 μM final concentration since the Km of this substrate was found to be 19.64 μM. Published methods were followed with minor modifications [79]. In brief: May1 active sites were titrated using the potent inhibitor peptstatin A and GraphPad Prism 6 software was used to determine May1 concentration from a plot of V_i_/V_o_ versus the log of inhibitor concentration.

Km was determined using 73 nM May1 (100 mM MES 100 mM NaCl pH 4.5) and 0.5 μM to 140 μM IQ-2. A correction factor was calculated to adjust for sensitivity of the plate reader by plotting the RFU value of complete cleavage versus the product concentration of IQ-2 from 0.5 μM—10 μM and dividing the V_max_ values from the Km calculation by 1/slope of this line, (units: RFU/[P]). Km was calculated by GraphPad Prism 6 software using the Michaelis-Menten equation.

IC_50_ calculations were conducted using 14.6 nM May1 (100 mM MES 100 mM NaCl pH 4.5). Inhibitor stocks were dissolved in DMSO and incubated with May1 for 10 minutes before addition of substrate. GraphPad Prism 6 was used to calculate IC_50_ values from a plot of the log of inhibitor concentration versus normalized response.

#### pH titration of May1 activity

Concentrated May1 was diluted to 14.6 nM in 100 mM MES, 100 mM NaCl buffers from pH 1.5–7. Fluorogenic activity assays were conducted using IQ-2 and the conditions described above.

### Macrophage studies

Bone-marrow derived macrophages (BMDMs) were isolated from C56Bl/6 mice and used for phagocytosis assays as described previously [80]. Briefly, BMDMs were plated in a 96-well plate (10,000/well) and simulated with 100 ng/ml Interferon-γ (Roche) starting 24 hr prior to assay initiation and continuing throughout. Overnight cultures of *C*. *neoformans* (14–16 hr) were grown in YNB media, after which cells were isolated, washed in DMEM and resuspended in BMDM growth media. Next, cells were opsonized with mAb1255 (10 μg/ml) at 37°C for 1 hr. *Cryptococcus* cells were added to macrophages at an MOI of 10, and this concentration was confirmed by plating yeast serial dilutions on rich media. After 24 hr at 37°C and 5% CO_2_, cells were washed three times with PBS to remove non-adherent yeast. Finally, ~200 BMDMs were quantified per well, with 6 wells per genotype, to determine the fraction of yeast-associated macrophages (phagocytic index).

*Cryptococcus* accumulation within macrophages was assessed as described previously [80]. Briefly, BMDMs were plated in 24 well plates at a concentration of 100,000 cells/well. Stimulation was performed as above, after which macrophages were exposed to opsonized *C*. *neoformans* at an MOI of 0.1. After 24 hr at 37°C and 5% CO_2_, supernatants were removed and macrophages were lysed. Serial dilutions were plated to determine CFU. The ratio of yeast present at 24 hr versus input was determined and analyzed by bootstrapping, generating 95% confidence intervals.

### Mouse virulence studies

*C*. *neoformans* strains were grown in liquid YNB cultures overnight (14–16 hr), and then centrifuged and washed twice in PBS. For competitive co-infection experiments, mixtures of a wild-type strain and a deletion strain of interest were prepared by determining cell concentration using a hemocytometer and then mixing strains in a 1:1 ratio to a final concentration of 1x10^7^ cells per ml PBS. Concentrations of viable cells were confirmed by plating serial dilutions. A/J female mice (Jackson Laboratory) aged 5–6 weeks were anesthetized by intraperitoneal injection of ketamine (75 mg/kg) and dexmedetomidine (0.5 mg/kg), then suspended from a silk thread by their front incisors, as described previously [81]. Intranasal infections of 50 μl were delivered by pipette, resulting in a dose of 5x10^5^ cells. After an additional 10 minutes of suspension, the mice were lowered and anesthesia reversed by intraperitoneal injection of atipamezole (1.5 mg/kg). Three mice were infected with each *C*. *neoformans* genotype, and were monitored until a defined terminal time point of ten days after infection. At this time, mice were sacrificed by CO_2_ inhalation and cervical spine dislocation. Next, lungs were harvested and homogenized in PBS using a PRO200 homogenizer (Grainger). The ratios of *C*. *neoformans* strains in the input and organ samples were determined by plating in serial dilutions on Sabouraud agar plates containing 40 mg/ml gentamicin and 50 mg/ml carbenicillin, and then testing the nourseothricin resistance status of ~200 colonies. As a negative control, mice were infected with a 1:1 ratio of wild-type cells and a *sxi1Δ* strain, which is known to have a wild-type phenotype [63].

For monotypic infections, female A/J mice were intranasally infected with 50 μl PBS containing *C*. *neoformans* cells of a single genotype at a concentration of 1.0x10^7^ cells per ml, as described above. Concentrations of viable cells were confirmed by plating serial dilutions. Ten mice were infected per genotype, and were monitored until severe morbidity (as indicated by a loss of 15% of initial body weight or other symptoms), at which point they were sacrificed. Survival data was analyzed using the Online Application for the Survival Analysis of Lifespan Assays Performed in Aging Research [82].

## Supporting Information

S1 Fig Fluorogenic assays indicate the endopeptidase class present in conditioned media and the optimal pH for detection of activity. (A) The impact of class specific peptidase inhibitors on peptidase activity. The class of enzyme inhibited by each compound is indicated in parenthesis. Averages and S.D. are shown for triplicates. The substrates cleaved most efficiently by peptidases in each media condition are shown (IQ-2 and IQ-7 for YNB and DMEM, respectively). Cleavage of the other IQ substrates by conditioned YNB media was also sensitive to pepstatin A, while cleavage of the other IQ substrates by DMEM conditioned media were also sensitive to 1-10-phenanthroline. (B) Screen of the effect of pH on proteolytic activity in YNB and DMEM supernatants. Three efficiently cleaved IQ substrates were chosen for this analysis. The activity scale is differs for this experiment because this assay was conducted on a SpectraMax Gemini plate reader (Molecular Devices) although conditions were otherwise equivalent. Averages and S.D. are shown for triplicates.
(PDF)

S2 Fig Reproducibility of MSP-MS assay. (A) YNB media conditioned by wild type *C*. *neoformans* was incubated with the 228-member MSP-MS peptide library for 15, 60, 240, and 1200 minutes. The number of cleavage sites was assessed at each time point, in triplicate. Error bars represent S.D. (B) Overlap of MSP-MS cleavage sites at the 1200 minute time point, among three replicates. (C-E) Substrate specificity profile of YNB media conditioned by wild type *C*. *neoformans*, as assessed in three technical replicates.
(PDF)

S3 Fig Functional categorization and analysis of secretion mechanism for proteomics results. (A) Functional categorization of all 24 proteins predicted to have a secretion signal. Functions were determined for unannotated proteins by the closest annotated protein after conducting a Blastp search. (B) Analysis of predicted secretion method for all proteins detected in YNB or DMEM conditioned media by proteomics.
(PDF)

S4 Fig MSP-MS analysis of secreted peptidase activity in *prb1Δ*, *CNAG_00150Δ*, *scx1Δ* and *cxd1Δ* strains cultured in DMEM. (A) Substrate specificity profiles of the serine peptidase deletion strains *prb1Δ* and *CNAG_00150Δ* and the carboxypeptidase deletion strains *scx1Δ* and *cxd1Δ* grown in DMEM, p < 0.05. (B) Positional analysis of the bonds cleaved in the four deletion strains. (C) Representative example of a peptide cleaved by peptidases in media conditioned by each of the four deletion strains.
(PDF)

S5 Fig MSP-MS analysis of secreted peptidase activity in *cxd2Δ*, *cxd3Δ* and *pep4Δ* strains cultured in YNB media. (A) Substrate specificity profiles of the carboxypeptidase deletion strains *cxd2Δ* and *cxd3Δ* as well as the aspartyl peptidase deletion strain *pep4Δ* grown in YNB, p < 0.05. (B) Positional analysis of the bonds cleaved in the four deletion strains. (C) An example of a representative peptide cleaved by conditioned media from each deletion strain.
(PDF)

S6 Fig IQ-2 is cleaved by May1. (A) Proteolysis of IQ-2 was measured in a fluorogenic assay of YNB supernatants from all peptidase deletion strains. Deletion of *MAY1* led to complete loss of cleavage of IQ-2. Columns represent mean ± S.D. (B) May1 was diluted to 14.6 nM in 100 mM MES pH 4.5, 100 mM NaCl and incubated with IQ-2. At the start of the reaction and after 24 hours of incubation at room temperature, samples were collected and analyzed by Matrix Assisted Laser Desorption Ionization-Time of Flight (MALDI-TOF). Based on analysis of its substrate specificity, it was hypothesized that May1 would cleave between the phenylalanine and leucine in IQ-2. The sodium adduct was observed for the N-terminal fragment of the expected cleavage product, confirming the site of cleavage.
(PDF)

S7 Fig Growth curves for all peptidase deletion strains. OD_600_ measurements were recorded for cultures grown in triplicate. Averages and S.D. of triplicates are shown.
(PDF)

S8 Fig Temperature and pH tolerance of peptidase deletion strains. (A) Two independent isolates of each peptidase deletion strain were spotted in a 10-fold dilution series on YNB agar plates and grown for 48 hours before imaging. (B) pH tolerance of *may1Δ* strains after 72 hours of growth.
(PDF)

S9 Fig Tolerance to solute, peroxide and cell wall stress and production of melanin of peptidase deletion strains. (A) 10-fold dilution series of all peptidase deletion strains were spotted on YNB agar plates containing the indicated stress and grown for 48 hours, except for H_2_O_2_ plates, which were grown for four days before imaging. (B) 10-fold dilution series of peptidase deletion strains grown on rich media plates (YPAD) containing 0.02% SDS and imaged after four days of growth. (C) Melanin production in the presence of L-DOPA. Strains were spotted in triplicate and images were taken after 72 hours of growth.
(PDF)

S10 Fig Screen of aspartyl peptidase inhibitors. Panels (A), (B) and (C) show the results of each inhibitor compound tested in triplicate at 100μM, 10μM and 1μM. The May1 activity against IQ-2 was measured. The average value and S.D. of triplicates are shown. (D) IC_50_ values were calculated for Brecanavir, pepstatin A and compounds 4, 16, 18 and 21. Values are averaged from triplicates and S.D. is shown by error bars.
(PDF)

S11 Fig May1 activity in cultures treated with aspartyl peptidase inhibitors. (A) Activity was recorded against the substrate IQ-2. Average values and S.D. of triplicate measurements are shown. (B) Density at saturation (after 48 hours of growth) is shown for YNB cultures of wild type or *may1Δ C*. *neoformans* treated with May1 inhibitors. Average values and S.D. of triplicates are shown.
(PDF)

S12 Fig Expression of genes neighboring *may1Δ*. (A-B) Transcript levels during conditions of low (A) or high (B) cell density in YNB medium, as assessed by RT-qPCR and normalized to 18S rRNA levels. Low density samples were harvested at a concentration of OD_600_ = 1.0, and high density samples were harvested after 32 hr of growth, as in conditioned media experiments. Average values and S.D. of duplicate samples are shown. (C) Map of *MAY1* locus, with indication of region deleted in *may1Δ* strains.
(PDF)

S13 Fig May1 is required for *C*. *neoformans* accumulation in macrophages. (A) Phagocytic index of opsonized *C*. *neoformans*. Error bars represent S.D. (B) Intracellular accumulation of *C*. *neoformans* in macrophages. * p < 0.05 versus wild type control. Error bars represent 95% confidence intervals.
(PDF)

S1 Table Sequences of internally quenched fluorogenic substrates. All peptides contain an N-terminal fluorophore: aminomethylcoumarin bound to the side chain of lysine or directly to the N-terminus as indicated, and a C-terminal quencher: di-nitrophenol bound to the side chain of lysine or directly to the C-terminus as indicated. “t” represents tert-butyl glycine and “n” represents norleucine.
(XLSX)

S2 Table Pearson correlations among of MSP-MS assay results and technical replicates. YNB media conditioned by wild type *C*. *neoformans* was incubated with the 228-member MSP-MS peptide library in three technical replicates. In each replicate, the frequency of every amino acid found at each of the 8 positions surrounding the cleaved bond was assessed. P4-P4' substrate specificity profiles were then created and compared using Pearson correlation. Correlation between the substrate specificity profiles of YNB media conditioned by wild type versus mutant strains was assessed in a similar manner.
(XLSX)

S3 Table Proteins identified by proteomics analysis. 32-hour YNB supernatants and 48-hour DMEM supernatants from wild-type *C*. *neoformans* cultures were analyzed. Since *C*. *neoformans* var *grubii* genes were not annotated in the version of the Uniprot database available, peptides were matched to proteins in other serotypes and the Uniprot accession numbers for *C*. *neoformans* var *grubii* proteins were then manually identified. “E value” stands for expectation value. An asterisk in the corresponding column indicates if a protein has a predicted secretion signal [44], is expected to be non-classically secreted [45] or has been associated with secreted microvesicles [46]. As indicated, after repeating proteomic analysis of the 32-hour YNB sample one additional peptidase was identified.
(XLSX)

S4 Table Expanded strain information. All strains used in this study are indicated, along with the CM number denoting their location in the Madhani laboratory strain database. “Nat^R^” indicates nourseothricin resistance, under the column labeled source “1” indicates a gift of J. Lodge, while “2” indicates strains created for this study.
(XLSX)

S5 Table Peptides observed by MSP-MS. All peptides detected by MSP-MS for each strain profiled are listed, as well as the reference set used to construct iceLogos from each dataset. An “n” is used to indicate norleucine, a replacement for methionine in the peptide library.
(XLSX)

S6 Table Doubling times and saturation densities of strains grown in YNB. Values shown are averages of triplicates grown in 25 mL YNB cultures. The online doubling time calculator was used to estimate doubling times during the exponential growth phase [78].
(XLSX)

S7 Table Aspartyl peptidase inhibitors. Structures of the 21 peptidomimetic aspartyl peptidase inhibitors used in this study as well as their effectiveness at inhibiting May1 activity at 1 μM concentration. Ten HIV protease inhibitors were also assessed.
(DOCX)
