# Supplementary figures and images for: Integrated Activity and Genetic Profiling of Secreted Peptidases in Cryptococcus neoformans Reveals an Aspartyl Peptidase Required for Low pH Survival and Virulence
Source: PLoS Pathog. 2016 Dec 15;12(12):e1006051. doi: 10.1371/journal.ppat.1006051 (PMC5158083; doi:10.1371/journal.ppat.1006051)

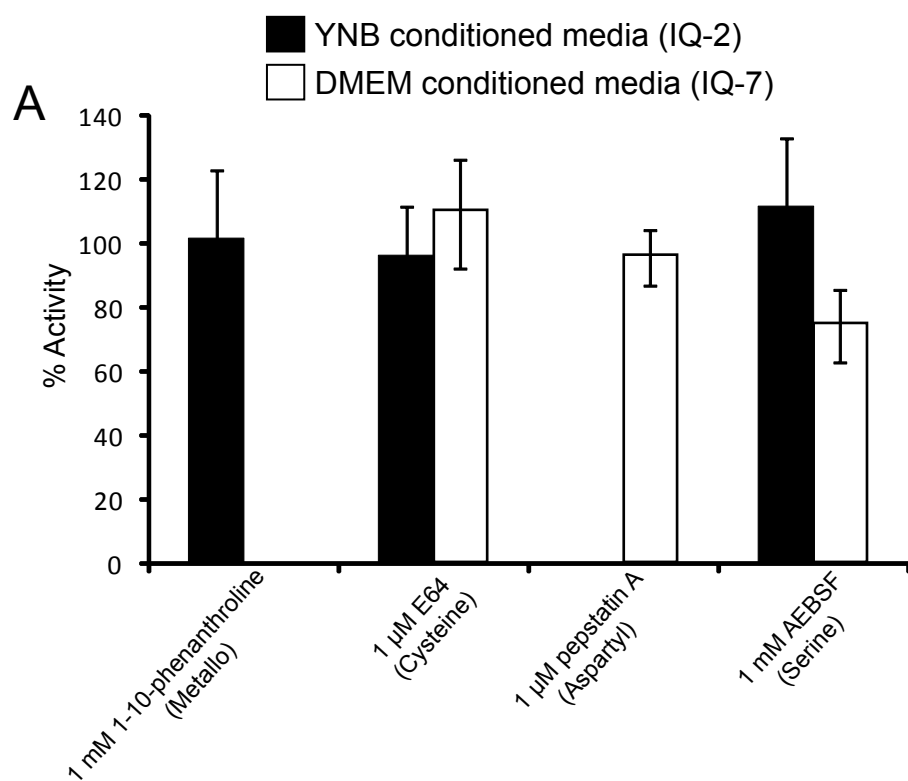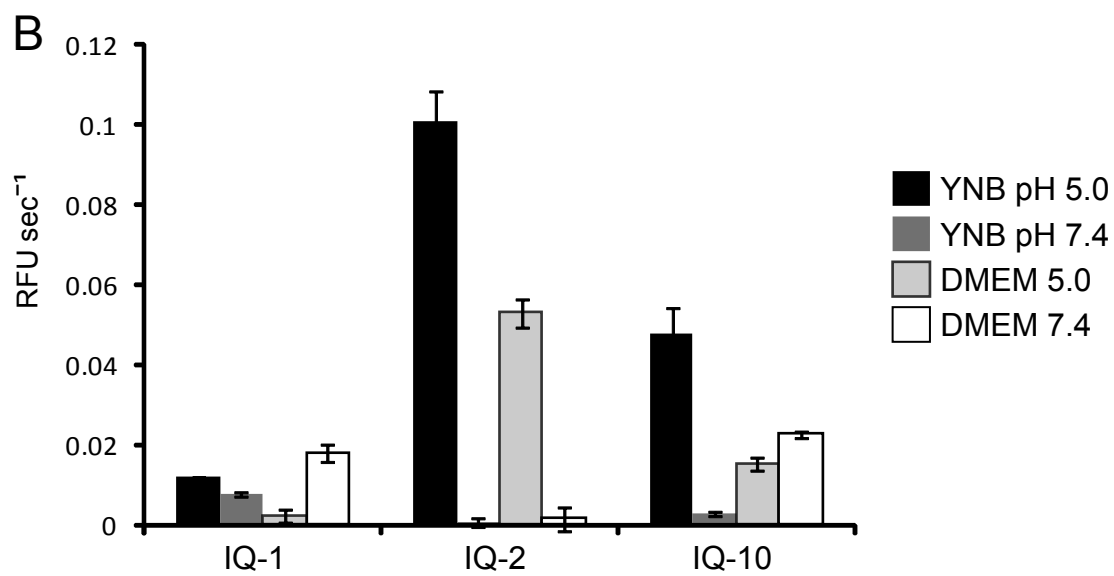

Supplement: S1 Fig — (A) The impact of class specific peptidase inhibitors on peptidase activity. The class of enzyme inhibited by each compound is indicated in parenthesis. Averages and S.D. are shown for triplicates. The substrates cleaved most efficiently by peptidases in each media condition are shown (IQ-2 and IQ-7 for YNB and DMEM, respectively). Cleavage of the other IQ substrates by conditioned YNB media was also sensitive to pepstatin A, while cleavage of the other IQ substrates by DMEM conditioned media were also sensitive to 1-10-phenanthroline. (B) Screen of the effect of pH on proteolytic activity in YNB and DMEM supernatants. Three efficiently cleaved IQ substrates were chosen for this analysis. The activity scale is differs for this experiment because this assay was conducted on a SpectraMax Gemini plate reader (Molecular Devices) although conditions were otherwise equivalent. Averages and S.D. are shown for triplicates. (PDF) [file ppat.1006051.s001.pdf]

**A**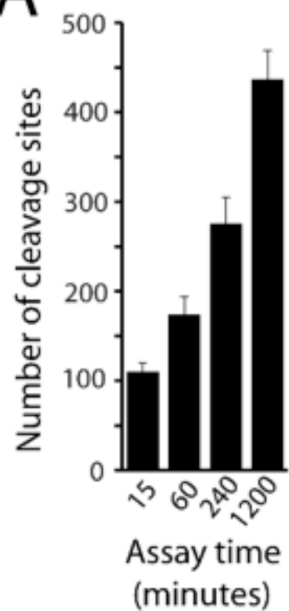**B**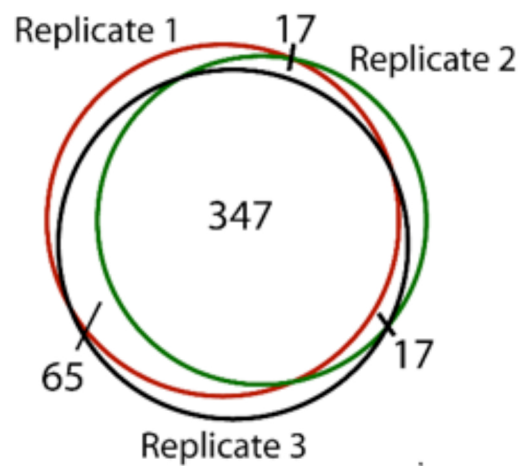**C**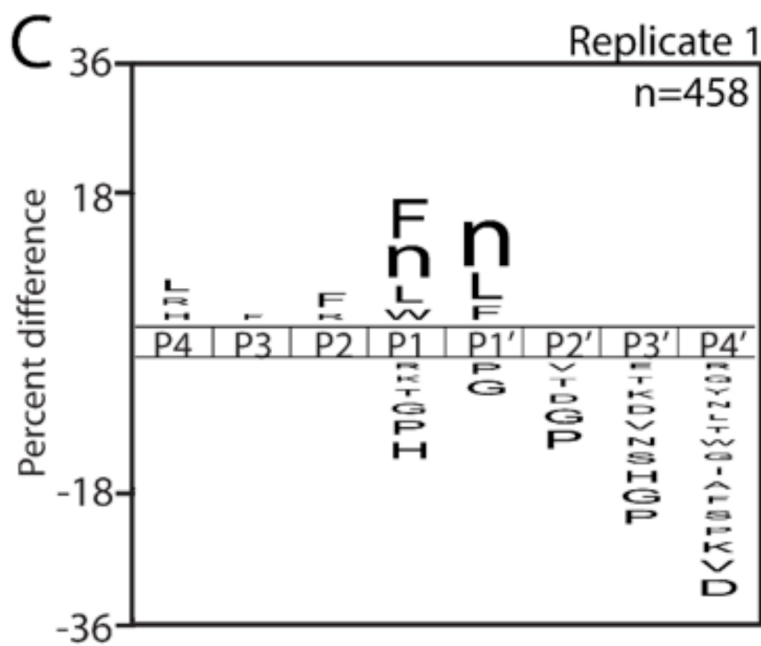**D**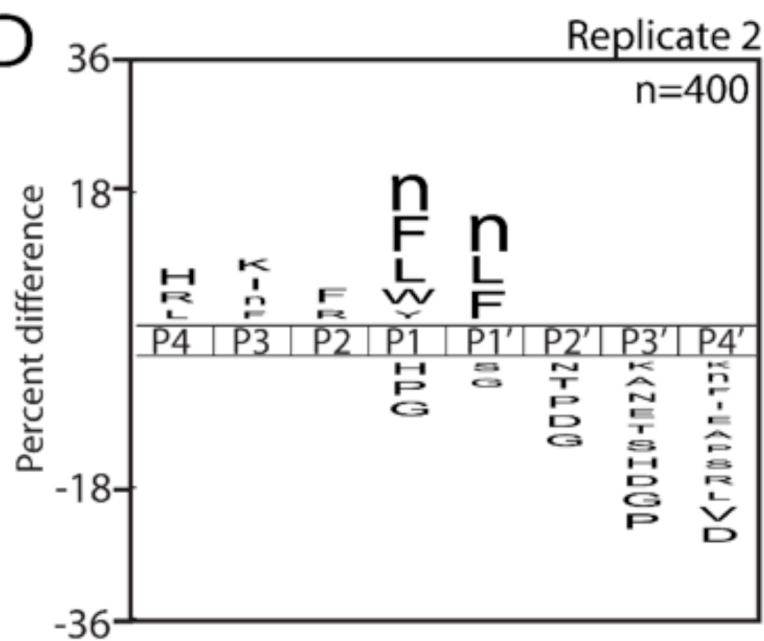**E**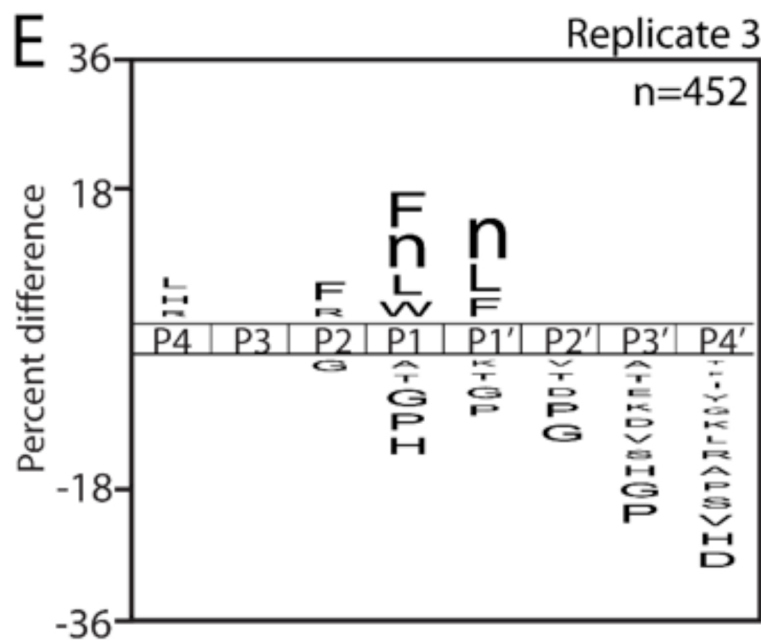

Supplement: S2 Fig — (A) YNB media conditioned by wild type C. neoformans was incubated with the 228-member MSP-MS peptide library for 15, 60, 240, and 1200 minutes. The number of cleavage sites was assessed at each time point, in triplicate. Error bars represent S.D. (B) Overlap of MSP-MS cleavage sites at the 1200 minute time point, among three replicates. (C-E) Substrate specificity profile of YNB media conditioned by wild type C. neoformans, as assessed in three technical replicates. (PDF) [file ppat.1006051.s002.pdf]

A

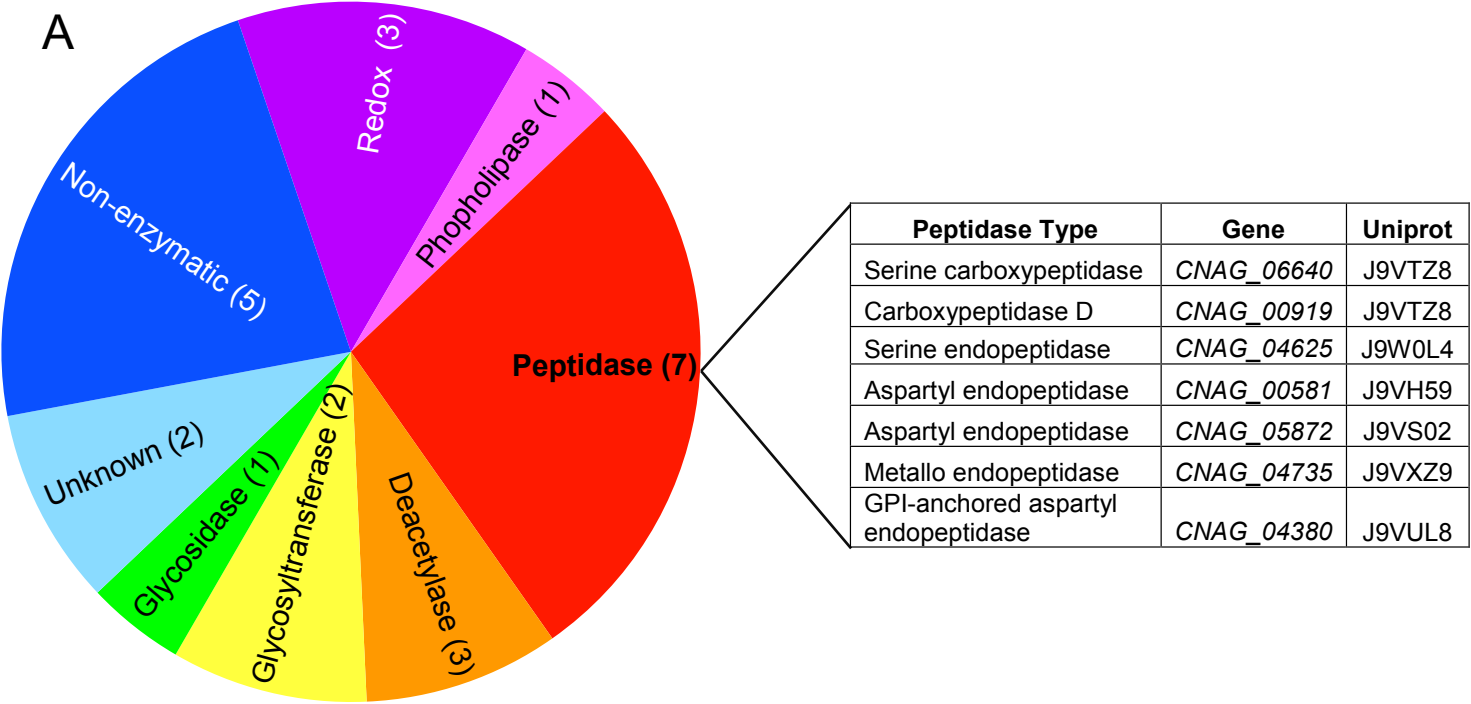

B

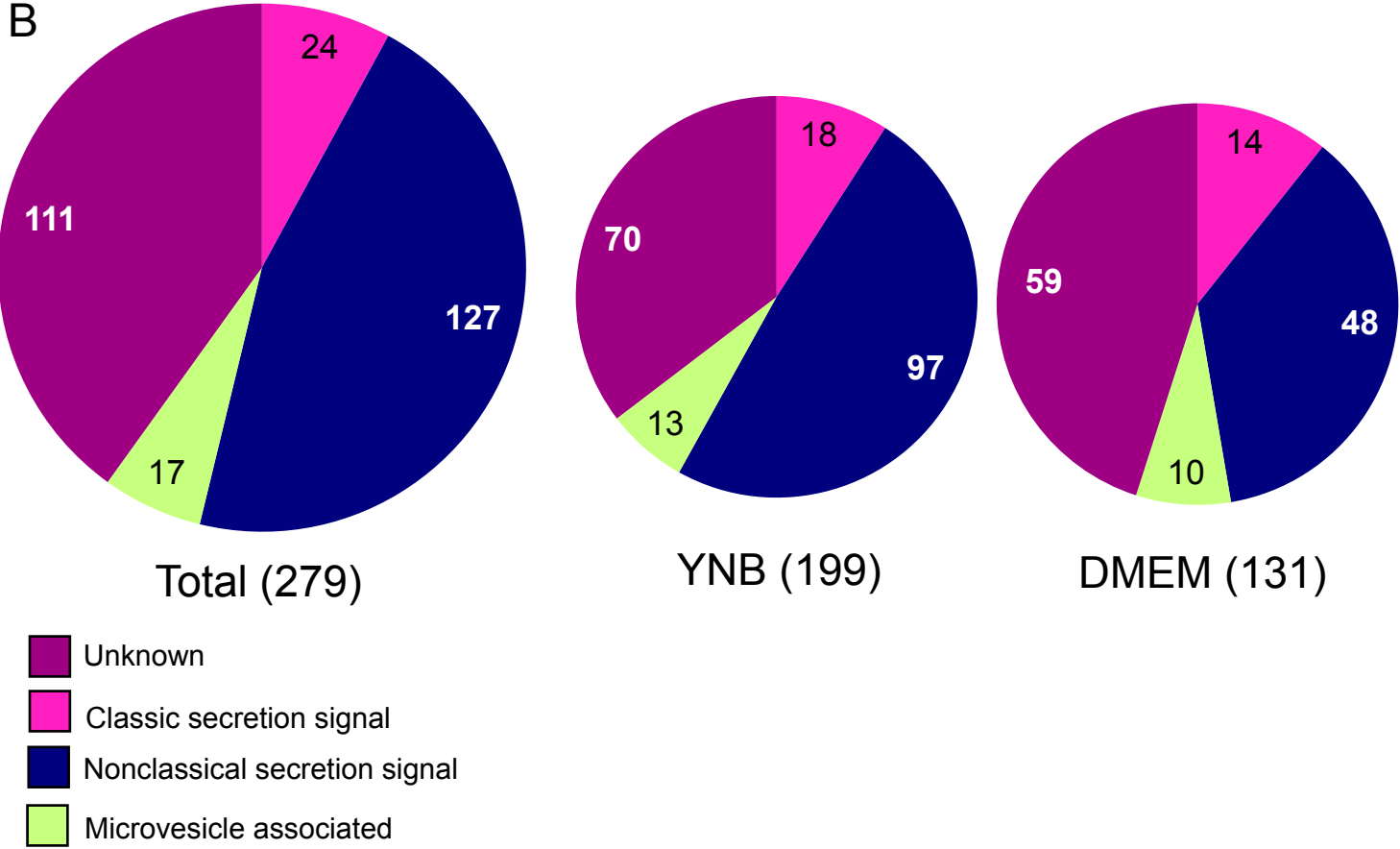

Supplement: S3 Fig — (A) Functional categorization of all 24 proteins predicted to have a secretion signal. Functions were determined for unannotated proteins by the closest annotated protein after conducting a Blastp search. (B) Analysis of predicted secretion method for all proteins detected in YNB or DMEM conditioned media by proteomics. (PDF) [file ppat.1006051.s003.pdf]

A

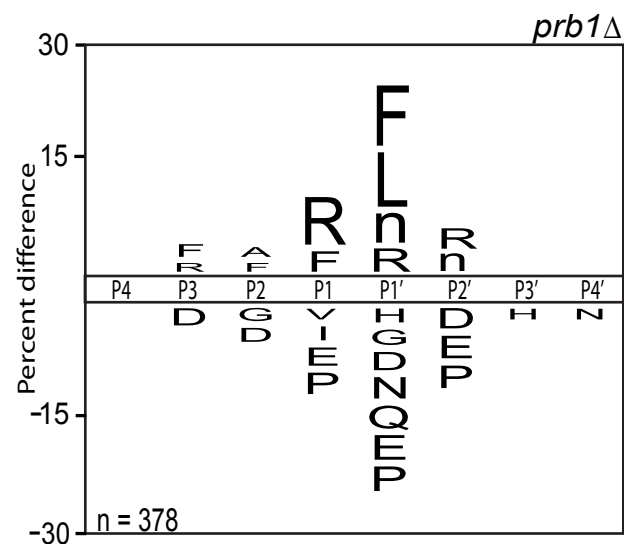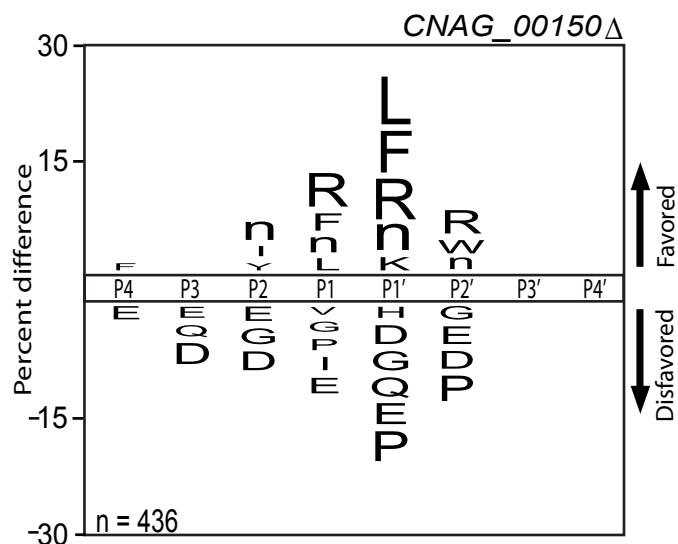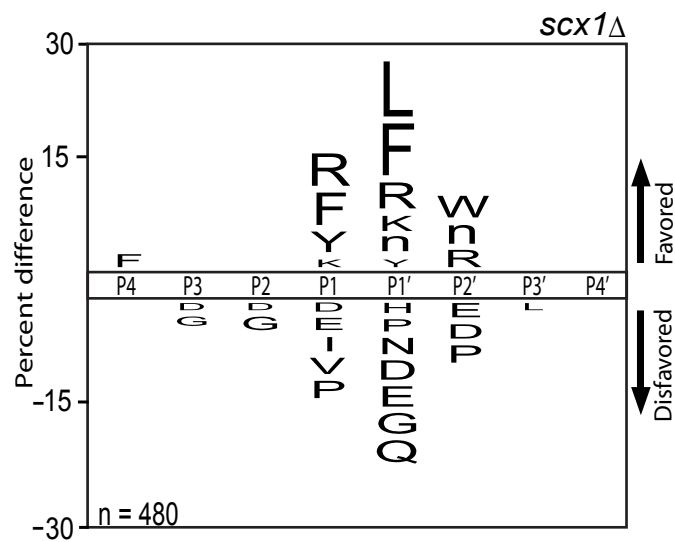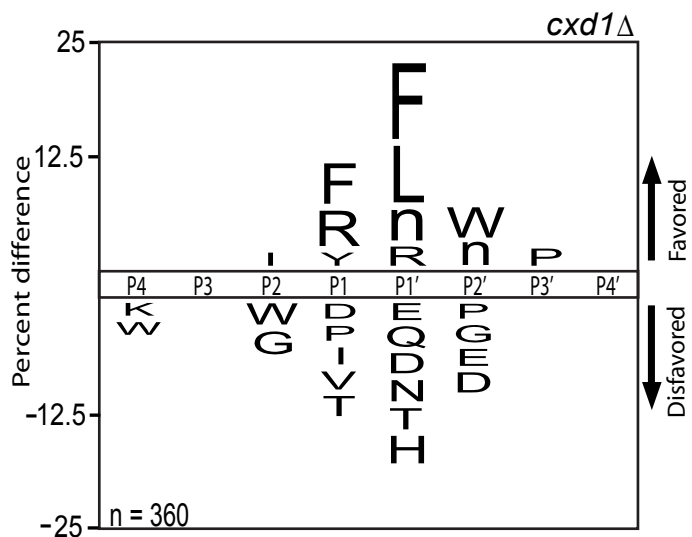

B

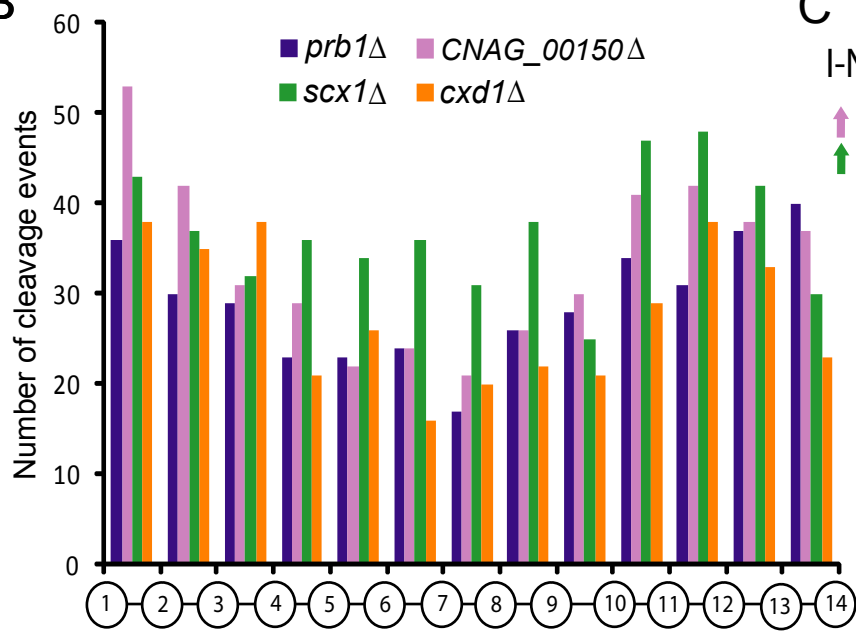

C

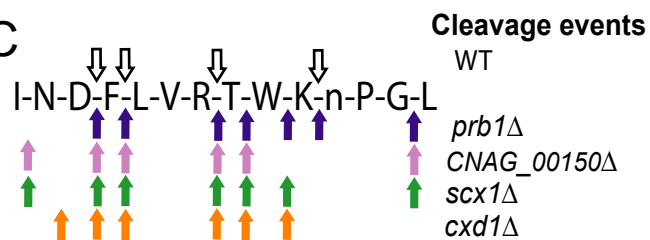

Supplement: S4 Fig — (A) Substrate specificity profiles of the serine peptidase deletion strains prb1Δ and CNAG_00150Δ and the carboxypeptidase deletion strains scx1Δ and cxd1Δ grown in DMEM, p < 0.05. (B) Positional analysis of the bonds cleaved in the four deletion strains. (C) Representative example of a peptide cleaved by peptidases in media conditioned by each of the four deletion strains. (PDF) [file ppat.1006051.s004.pdf]

A

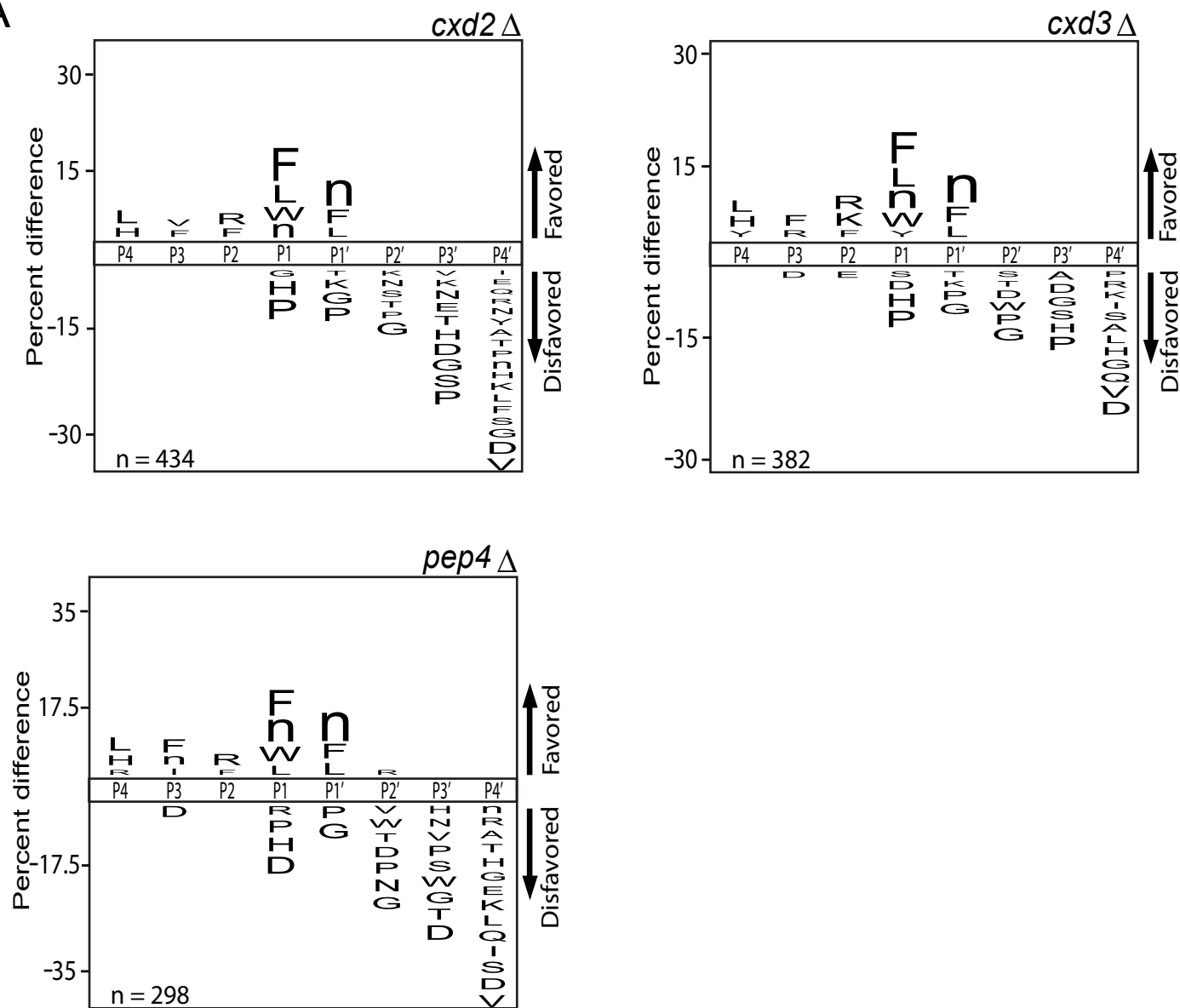

B

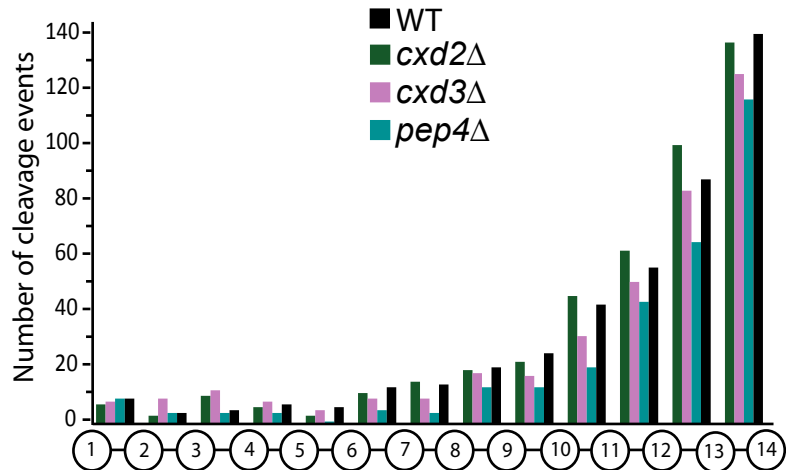

C

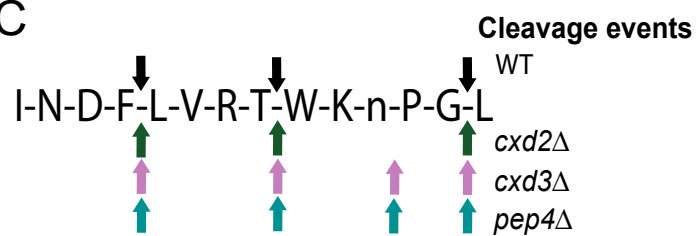

Supplement: S5 Fig — (A) Substrate specificity profiles of the carboxypeptidase deletion strains cxd2Δ and cxd3Δ as well as the aspartyl peptidase deletion strain pep4Δ grown in YNB, p < 0.05. (B) Positional analysis of the bonds cleaved in the four deletion strains. (C) An example of a representative peptide cleaved by conditioned media from each deletion strain. (PDF) [file ppat.1006051.s005.pdf]

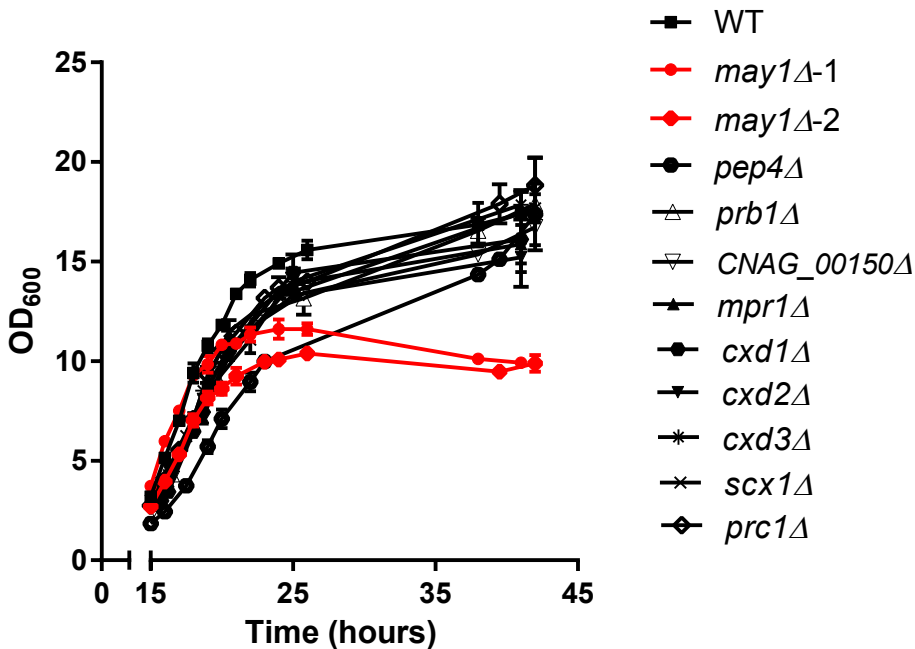

Supplement: S7 Fig — OD600 measurements were recorded for cultures grown in triplicate. Averages and S.D. of triplicates are shown. (PDF) [file ppat.1006051.s007.pdf]

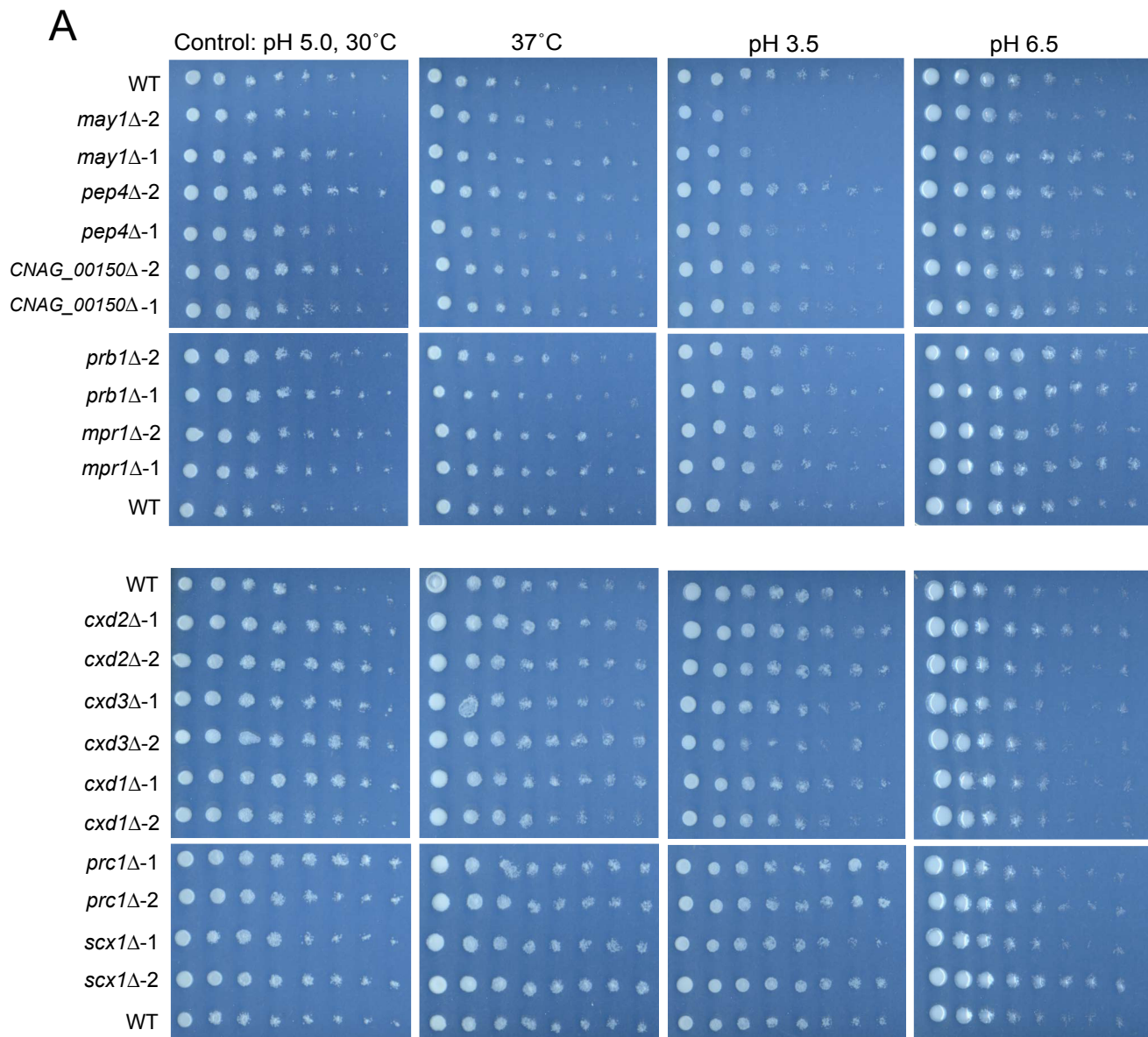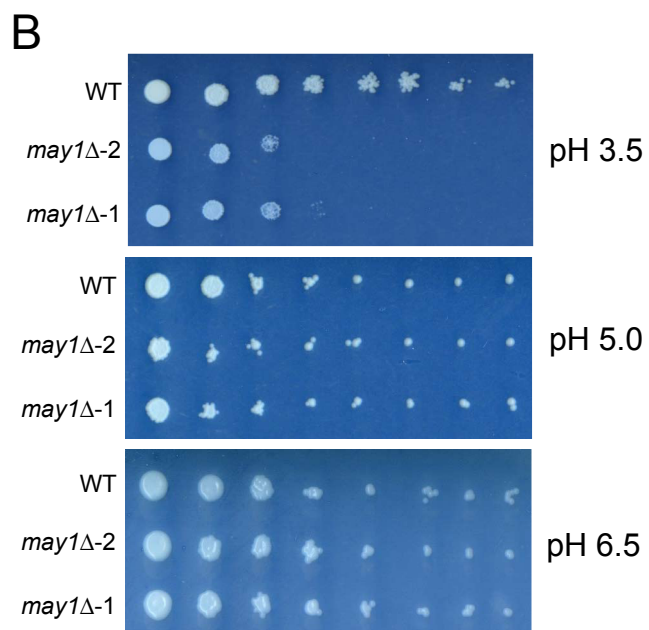

Supplement: S8 Fig — (A) Two independent isolates of each peptidase deletion strain were spotted in a 10-fold dilution series on YNB agar plates and grown for 48 hours before imaging. (B) pH tolerance of may1Δ strains after 72 hours of growth. (PDF) [file ppat.1006051.s008.pdf]

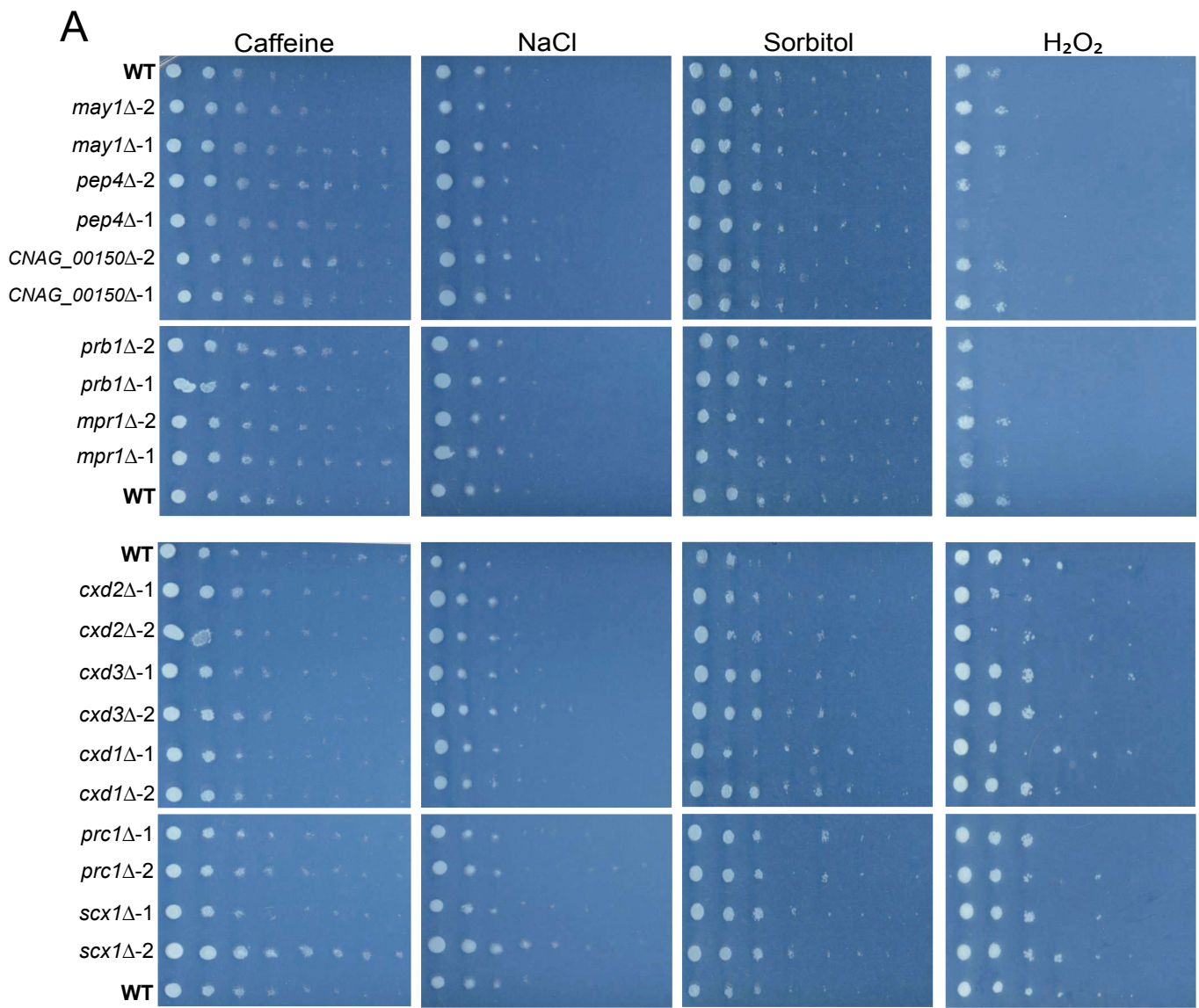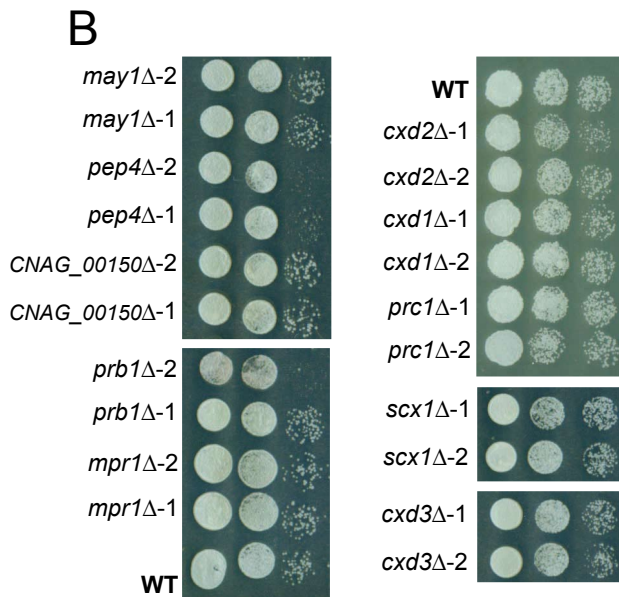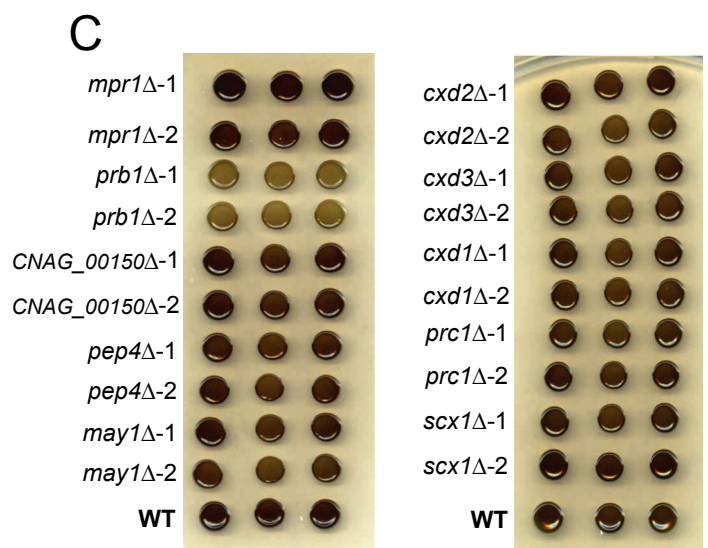

Supplement: S9 Fig — (A) 10-fold dilution series of all peptidase deletion strains were spotted on YNB agar plates containing the indicated stress and grown for 48 hours, except for H2O2 plates, which were grown for four days before imaging. (B) 10-fold dilution series of peptidase deletion strains grown on rich media plates (YPAD) containing 0.02% SDS and imaged after four days of growth. (C) Melanin production in the presence of L-DOPA. Strains were spotted in triplicate and images were taken after 72 hours of growth. (PDF) [file ppat.1006051.s009.pdf]

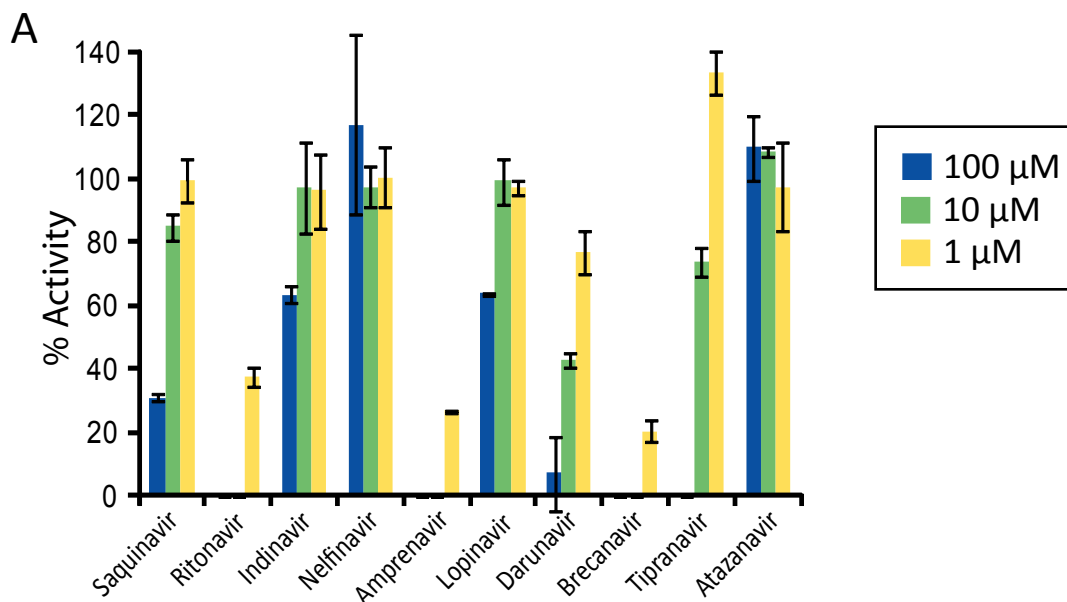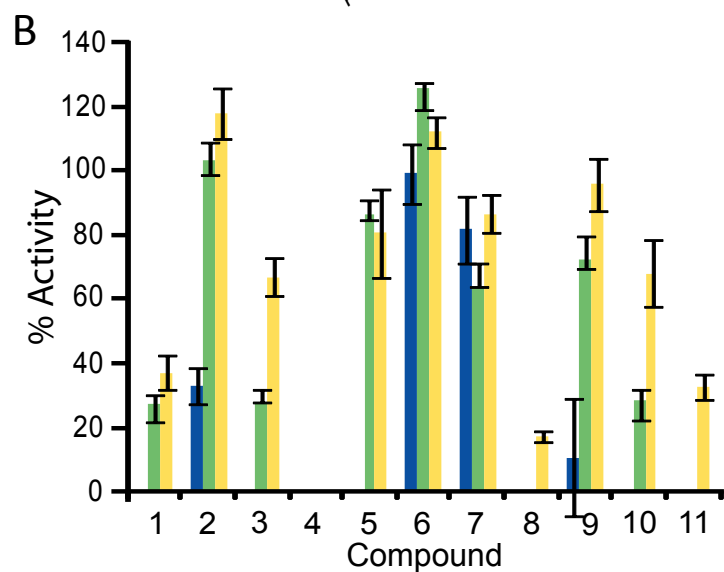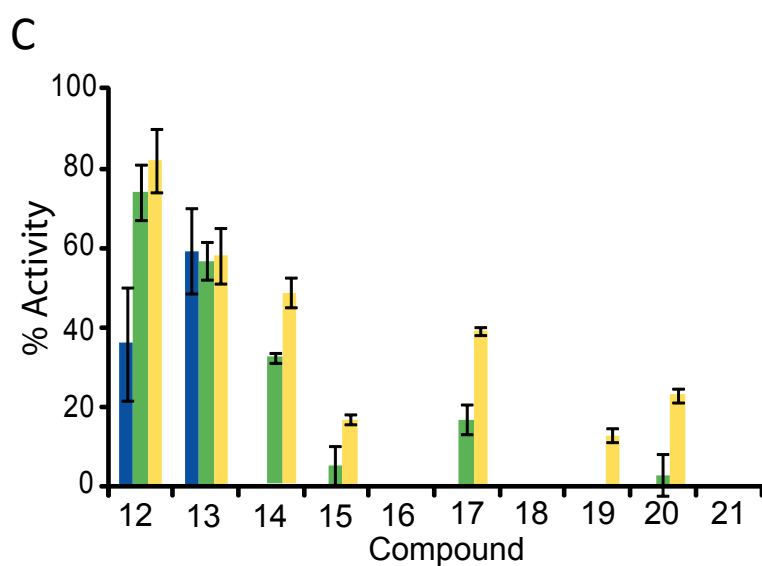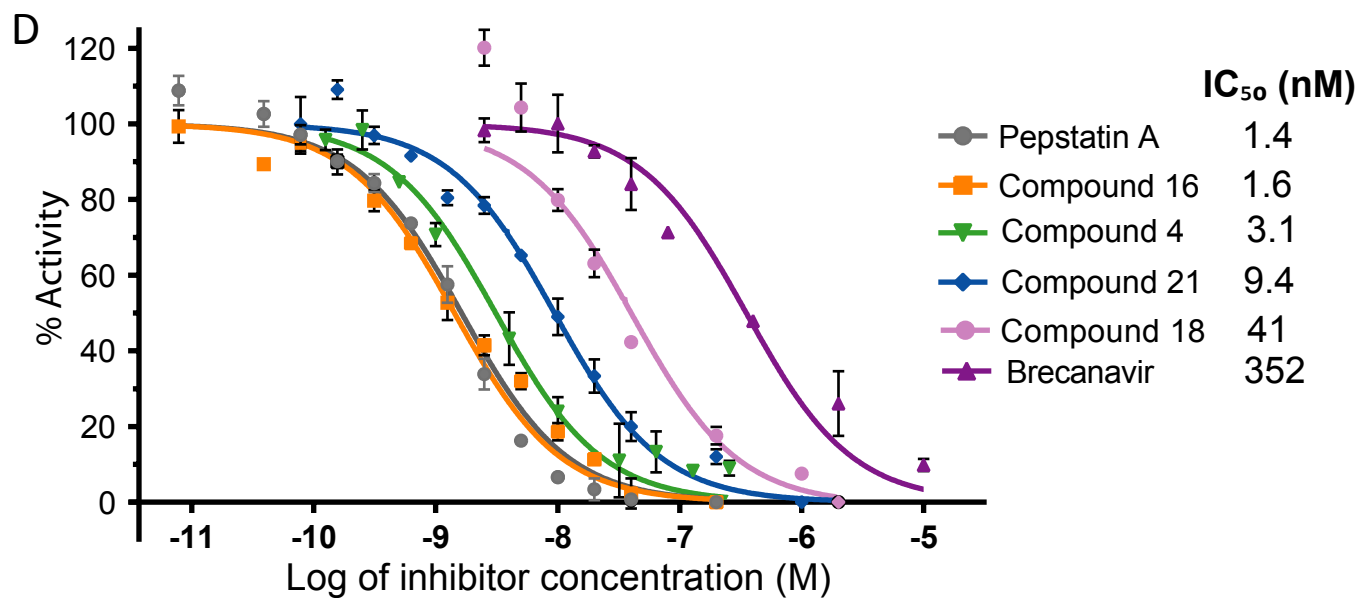

Supplement: S10 Fig — Panels (A), (B) and (C) show the results of each inhibitor compound tested in triplicate at 100μM, 10μM and 1μM. The May1 activity against IQ-2 was measured. The average value and S.D. of triplicates are shown. (D) IC50 values were calculated for Brecanavir, pepstatin A and compounds 4, 16, 18 and 21. Values are averaged from triplicates and S.D. is shown by error bars. (PDF) [file ppat.1006051.s010.pdf]

A

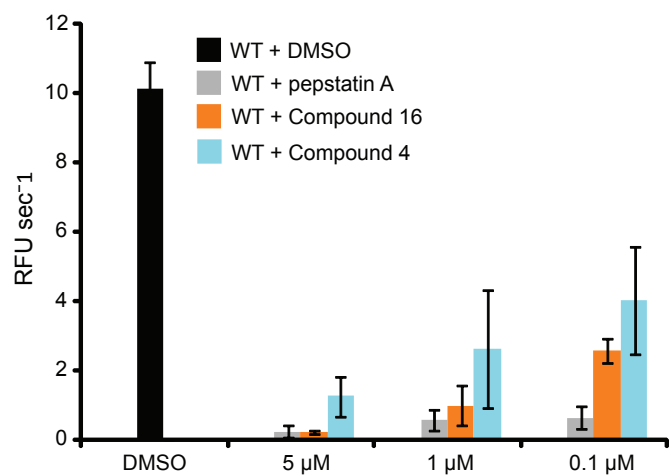

B

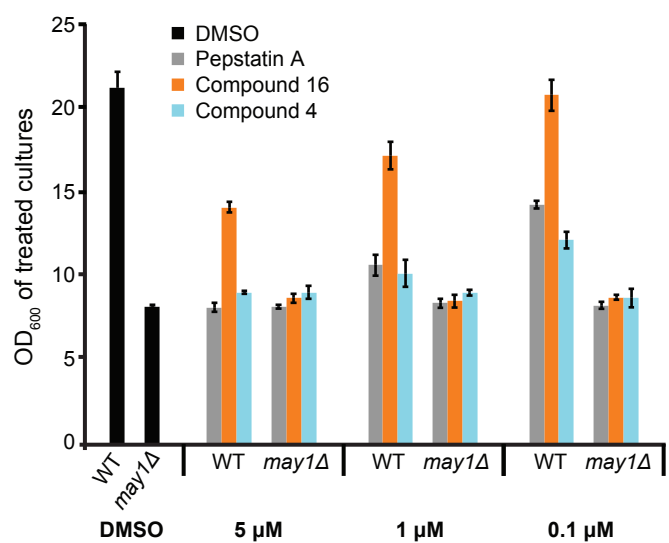

Supplement: S11 Fig — (A) Activity was recorded against the substrate IQ-2. Average values and S.D. of triplicate measurements are shown. (B) Density at saturation (after 48 hours of growth) is shown for YNB cultures of wild type or may1Δ C. neoformans treated with May1 inhibitors. Average values and S.D. of triplicates are shown. (PDF) [file ppat.1006051.s011.pdf]

A

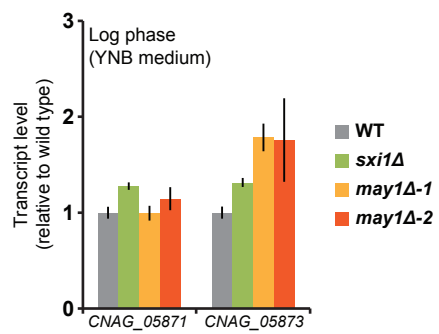

B

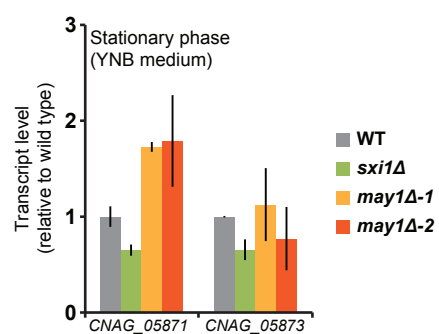

C

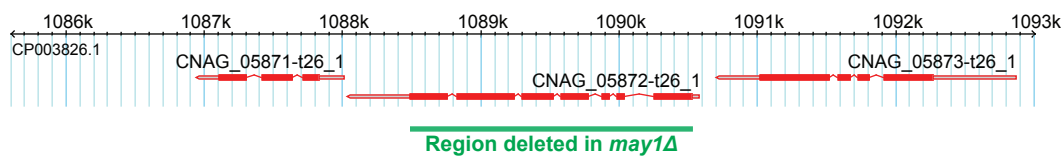

Supplement: S12 Fig — (A-B) Transcript levels during conditions of low (A) or high (B) cell density in YNB medium, as assessed by RT-qPCR and normalized to 18S rRNA levels. Low density samples were harvested at a concentration of OD600 = 1.0, and high density samples were harvested after 32 hr of growth, as in conditioned media experiments. Average values and S.D. of duplicate samples are shown. (C) Map of MAY1 locus, with indication of region deleted in may1Δ strains. (PDF) [file ppat.1006051.s012.pdf]

**A**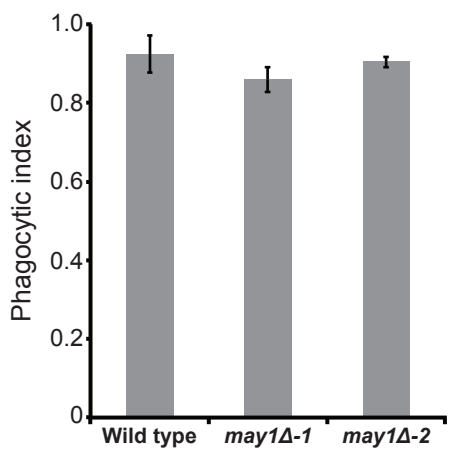**B**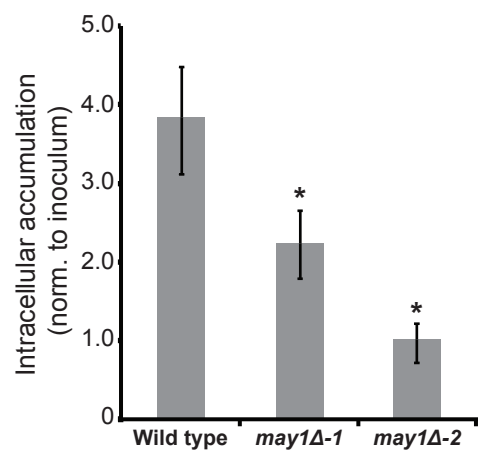

Supplement: S13 Fig — (A) Phagocytic index of opsonized C. neoformans. Error bars represent S.D. (B) Intracellular accumulation of C. neoformans in macrophages. * p < 0.05 versus wild type control. Error bars represent 95% confidence intervals. (PDF) [file ppat.1006051.s013.pdf]
