# Supplementary material for: Integrated Activity and Genetic Profiling of Secreted Peptidases in Cryptococcus neoformans Reveals an Aspartyl Peptidase Required for Low pH Survival and Virulence
Source: PLoS Pathog. 2016 Dec 15;12(12):e1006051. doi: 10.1371/journal.ppat.1006051 (PMC5158083; doi:10.1371/journal.ppat.1006051)
Supplement: S6 Fig — (A) Proteolysis of IQ-2 was measured in a fluorogenic assay of YNB supernatants from all peptidase deletion strains. Deletion of MAY1 led to complete loss of cleavage of IQ-2. Columns represent mean ± S.D. (B) May1 was diluted to 14.6 nM in 100 mM MES pH 4.5, 100 mM NaCl and incubated with IQ-2. At the start of the reaction and after 24 hours of incubation at room temperature, samples were collected and analyzed by Matrix Assisted Laser Desorption Ionization-Time of Flight (MALDI-TOF). Based on analysis of its substrate specificity, it was hypothesized that May1 would cleave between the phenylalanine and leucine in IQ-2. The sodium adduct was observed for the N-terminal fragment of the expected cleavage product, confirming the site of cleavage. (PDF) [file ppat.1006051.s006.pdf]

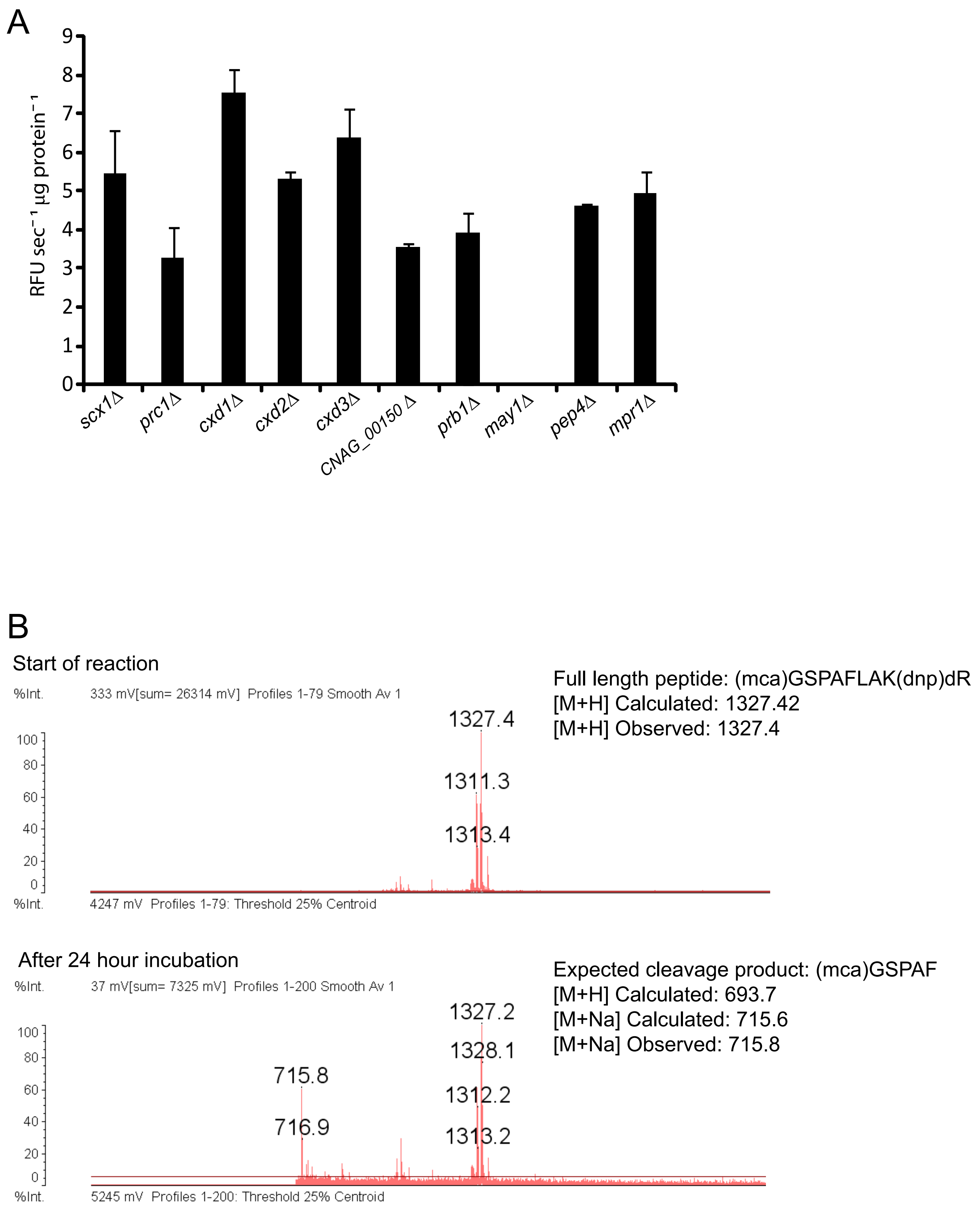
A
RFU sec⁻¹ µg protein⁻¹
0
1
2
3
4
5
6
7
8
9
scx1Δ
prc1Δ
cxd1Δ
cxd2Δ
cxd3Δ
CNAG_00150 Δ
prb1Δ
may1Δ
pep4Δ
mpr1Δ
B
Start of reaction
%Int.
333 mV[sum= 26314 mV] Profiles 1-79 Smooth Av 1
1327.4
1311.3
1313.4
100
80
60
40
20
0
%Int.
4247 mV Profiles 1-79: Threshold 25% Centroid
Full length peptide: (mca)GSPAFLAK(dnp)dR
[M+H] Calculated: 1327.42
[M+H] Observed: 1327.4
After 24 hour incubation
%Int.
37 mV[sum= 7325 mV] Profiles 1-200 Smooth Av 1
1327.2
1328.1
715.8
1312.2
716.9
1313.2
100
80
60
40
20
0
%Int.
5245 mV Profiles 1-200: Threshold 25% Centroid
Expected cleavage product: (mca)GSPAF
[M+H] Calculated: 693.7
[M+Na] Calculated: 715.6
[M+Na] Observed: 715.8
