# Supplementary material for: Integrated Activity and Genetic Profiling of Secreted Peptidases in Cryptococcus neoformans Reveals an Aspartyl Peptidase Required for Low pH Survival and Virulence
Source: PLoS Pathog. 2016 Dec 15;12(12):e1006051. doi: 10.1371/journal.ppat.1006051 (PMC5158083; doi:10.1371/journal.ppat.1006051)
Supplement: S7 Table — Structures of the 21 peptidomimetic aspartyl peptidase inhibitors used in this study as well as their effectiveness at inhibiting May1 activity at 1 μM concentration. Ten HIV protease inhibitors were also assessed. (DOCX) [file ppat.1006051.s020.docx]

| **S7 Table. Structures of aspartyl peptidase inhibitors** | | |
| --- | --- | --- |
| **Compound** | **Structure** | **% May1 inhibition at 1 uM** |
| **1** |  | 62 |
| **2** |  | 0 |
| **3** |  | 33 |
| **4** |  | 100 |
| **5** |  | 19 |
| **6** |  | 0 |
| **7** |  | 13 |
| **8** |  | 83 |
| **9** |  | 4 |
| **10** |  | 32 |
| **11** |  | 68 |
| **12** |  | 18 |
| **13** |  | 42 |
| **14** |  | 52 |
| **15** |  | 83 |
| **16** |  | 100 |
| **17** |  | 61 |
| **18** |  | 100 |
| **19** |  | 87 |
| **20** |  | 77 |
| **21** |  | 100 |
